# Supplementary material for: Immunometabolic Markers in a Small Patient Cohort Undergoing Immunotherapy
Source: Biomolecules. 2022 May 18;12(5):716. doi: 10.3390/biom12050716 (PMC9139165; doi:10.3390/biom12050716)
Supplement: Supplementary file 1 [file biomolecules-12-00716-s001.zip › Supplemental Data 1 FACS Gating.pptx]

## Slide 1
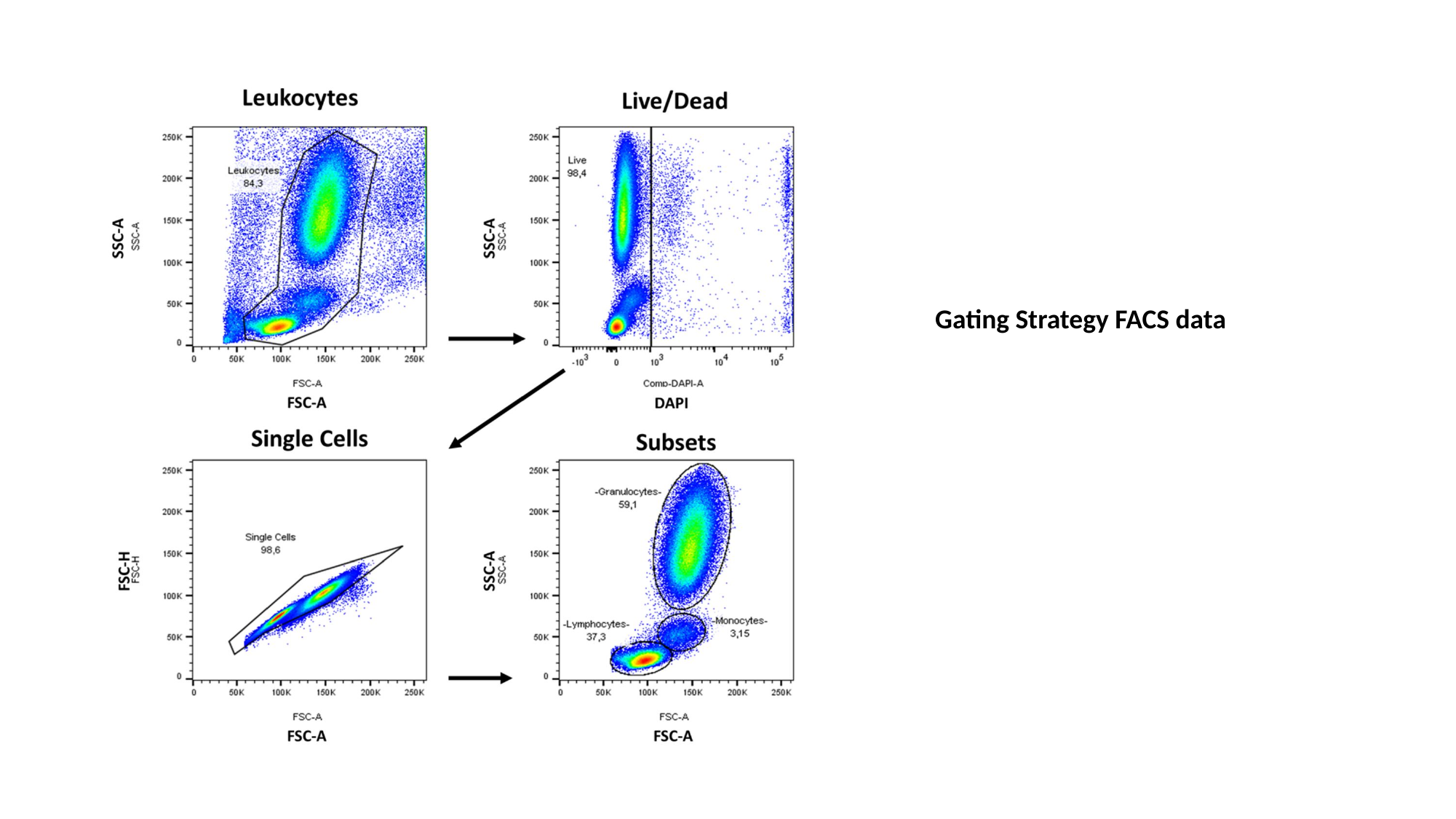

Gating Strategy FACS data

## Slide 2
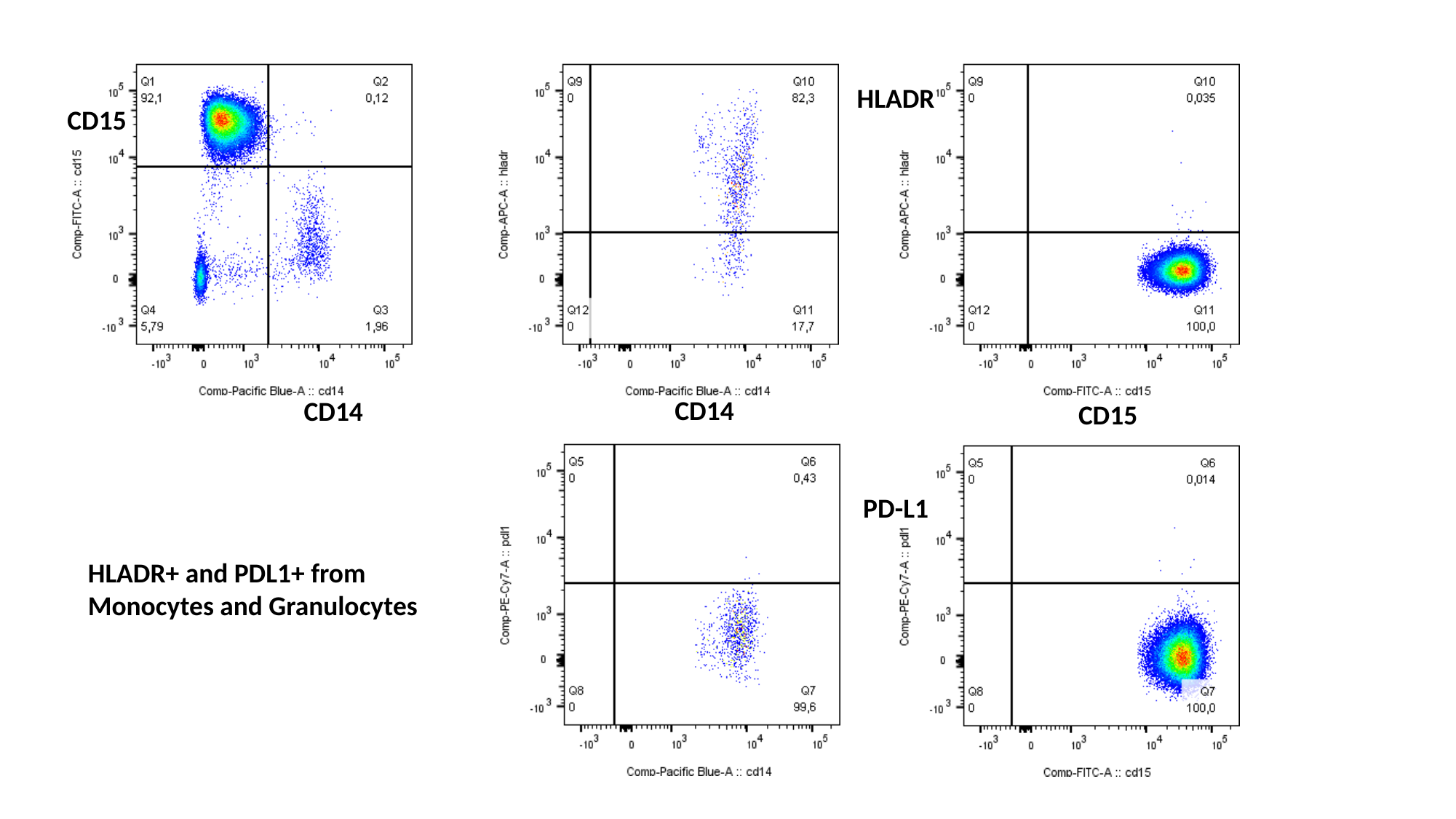

HLADR
CD15
CD14
CD14
CD15
PD-L1
HLADR+ and PDL1+ from
Monocytes and Granulocytes

## Slide 3
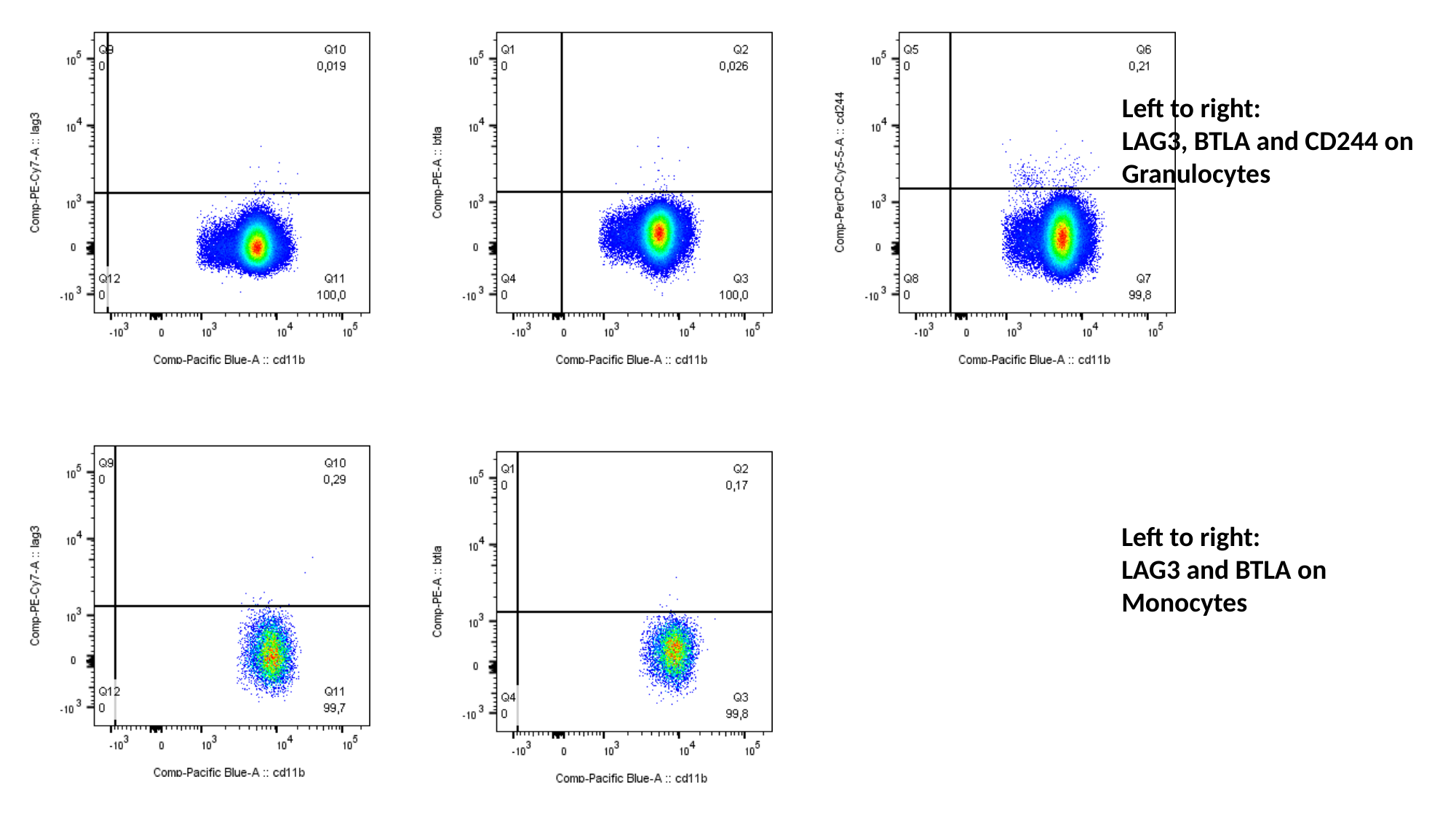

Left to right:
LAG3, BTLA and CD244 on
Granulocytes
Left to right:
LAG3 and BTLA on
Monocytes

## Slide 4
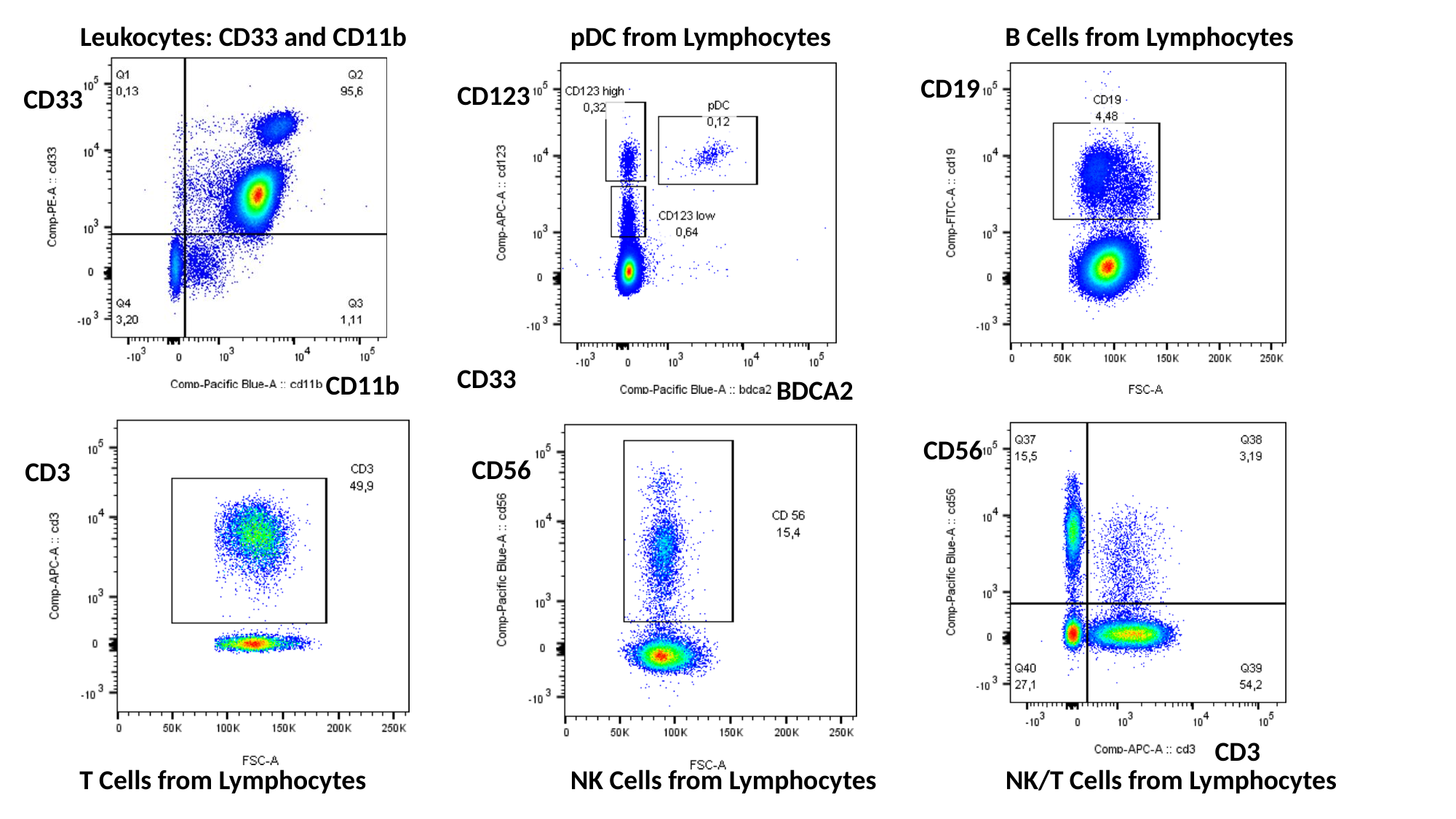

Leukocytes: CD33 and CD11b
pDC from Lymphocytes
B Cells from Lymphocytes
T Cells from Lymphocytes
NK Cells from Lymphocytes
NK/T Cells from Lymphocytes
CD19
CD123
CD33
CD33
CD11b
BDCA2
CD56
CD56
CD3
CD3

## Slide 5
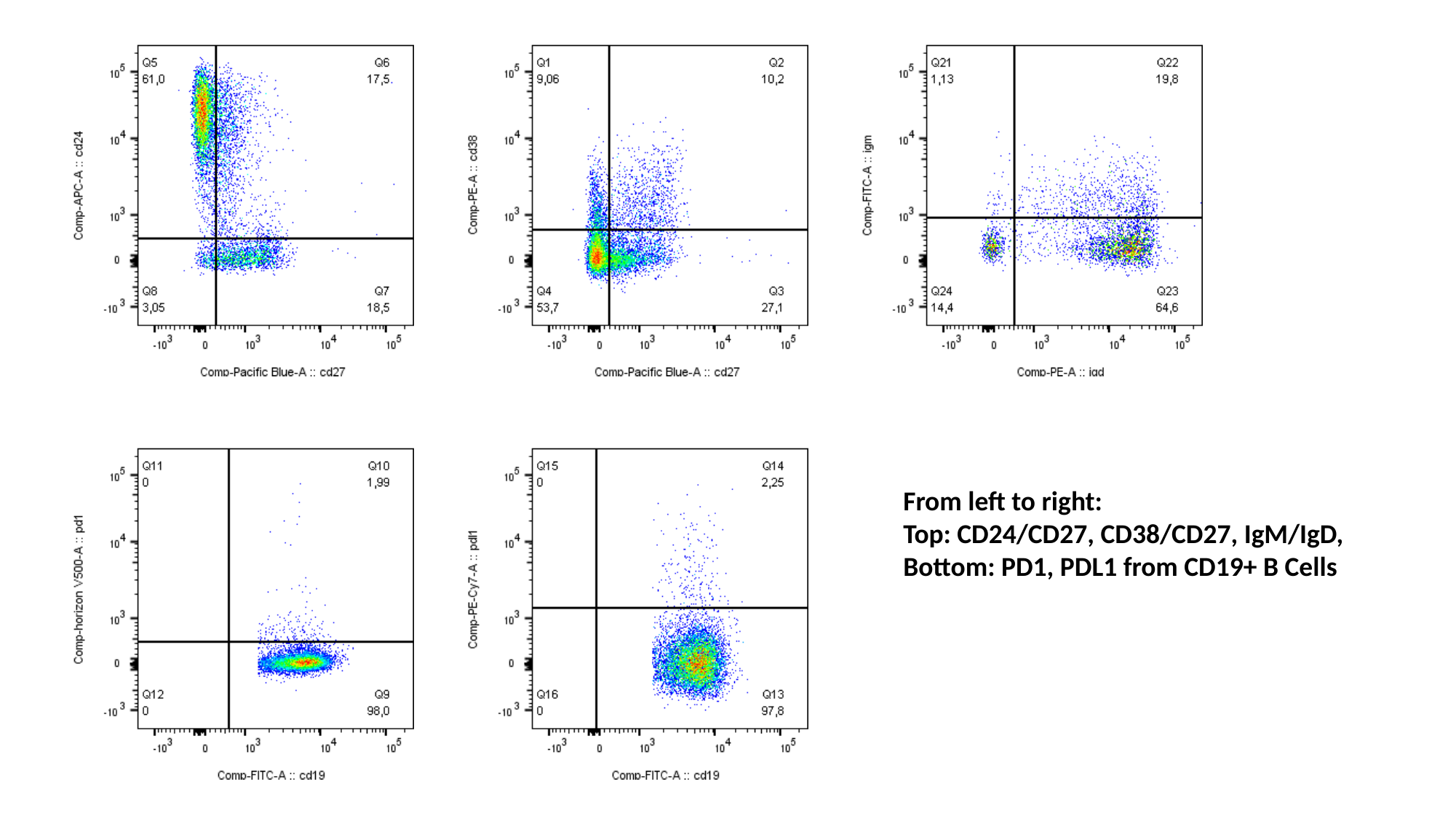

From left to right:
Top: CD24/CD27, CD38/CD27, IgM/IgD,
Bottom: PD1, PDL1 from CD19+ B Cells

## Slide 6
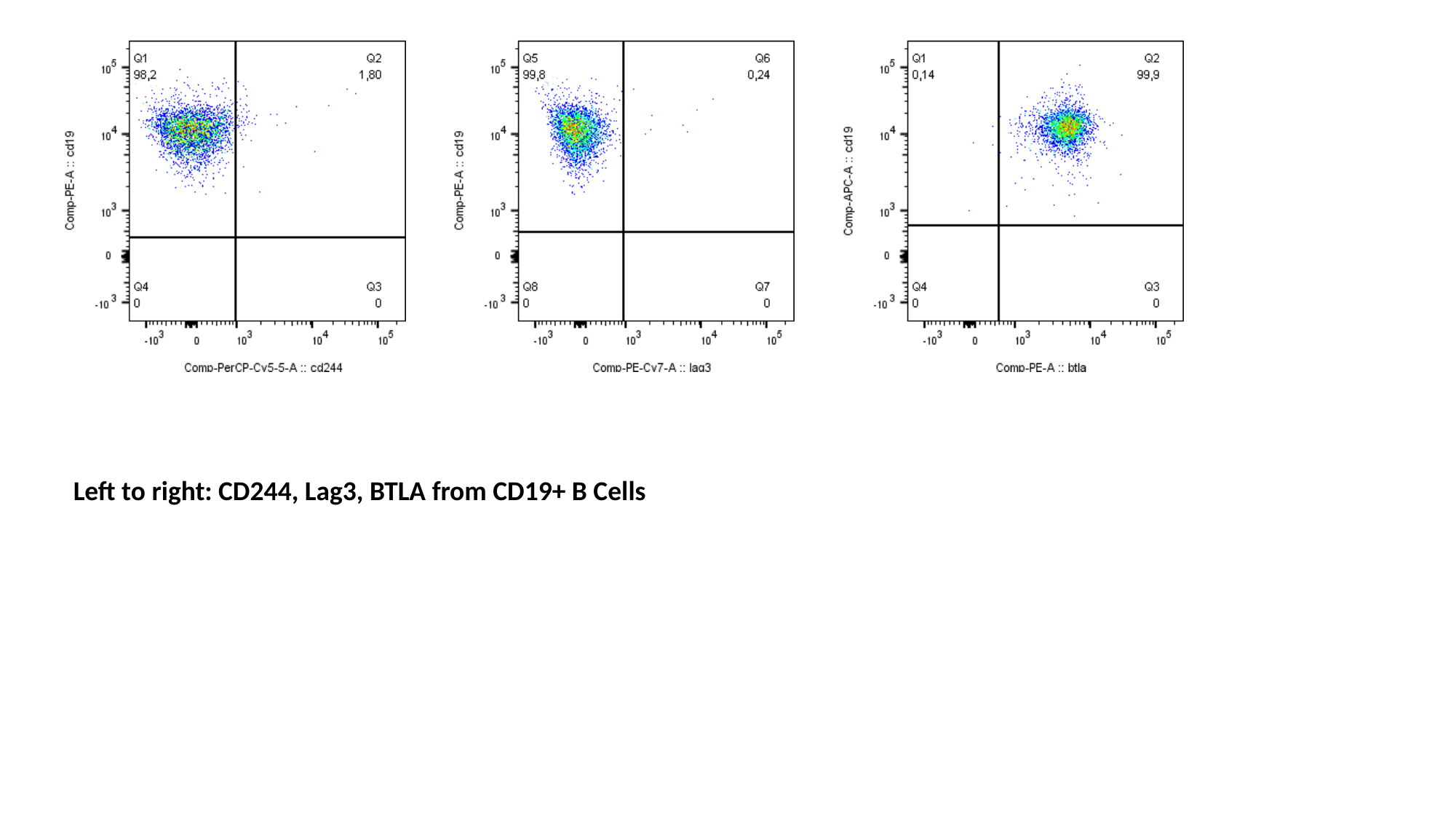

Left to right: CD244, Lag3, BTLA from CD19+ B Cells

## Slide 7
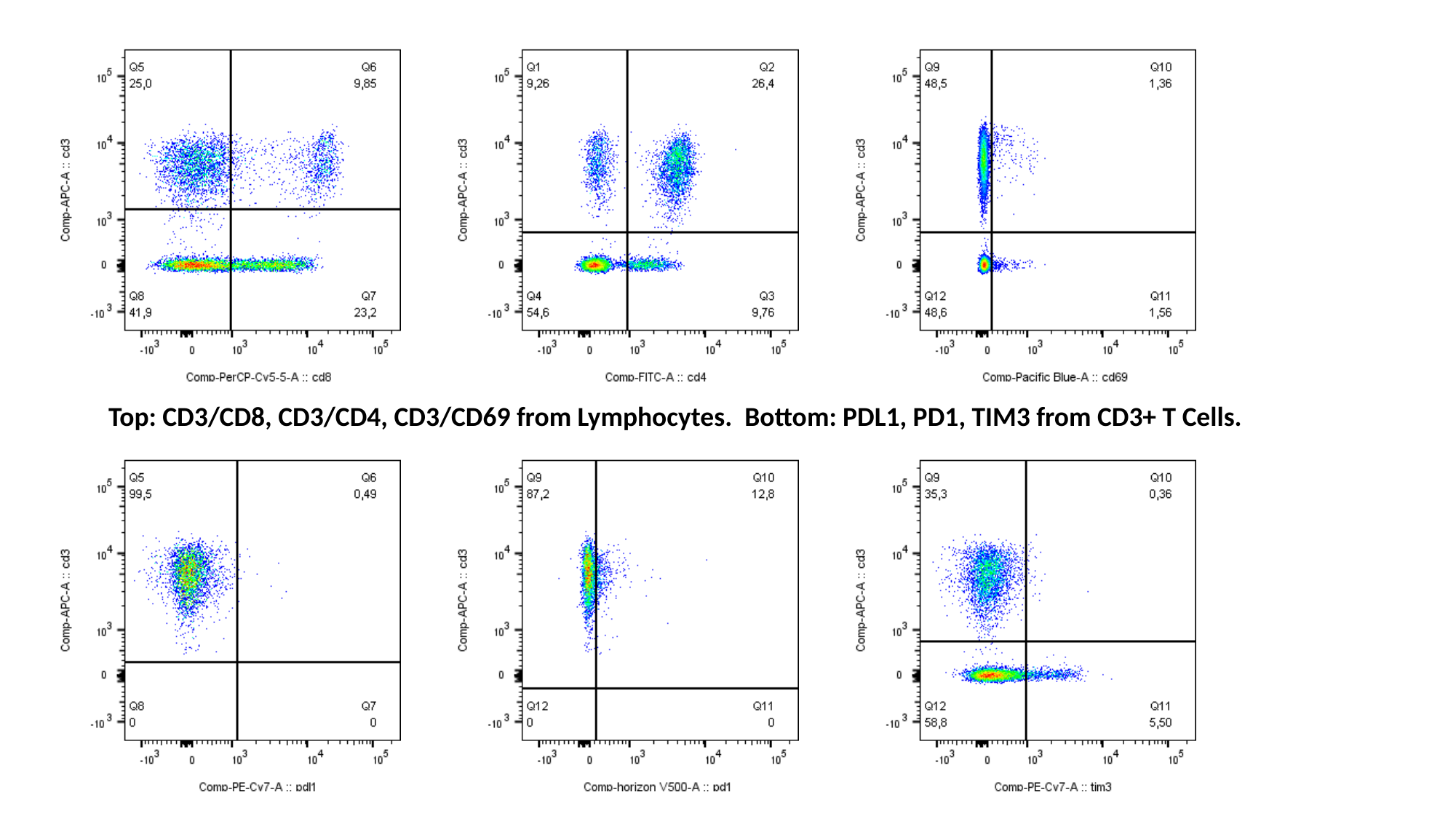

Top: CD3/CD8, CD3/CD4, CD3/CD69 from Lymphocytes. Bottom: PDL1, PD1, TIM3 from CD3+ T Cells.

## Slide 8
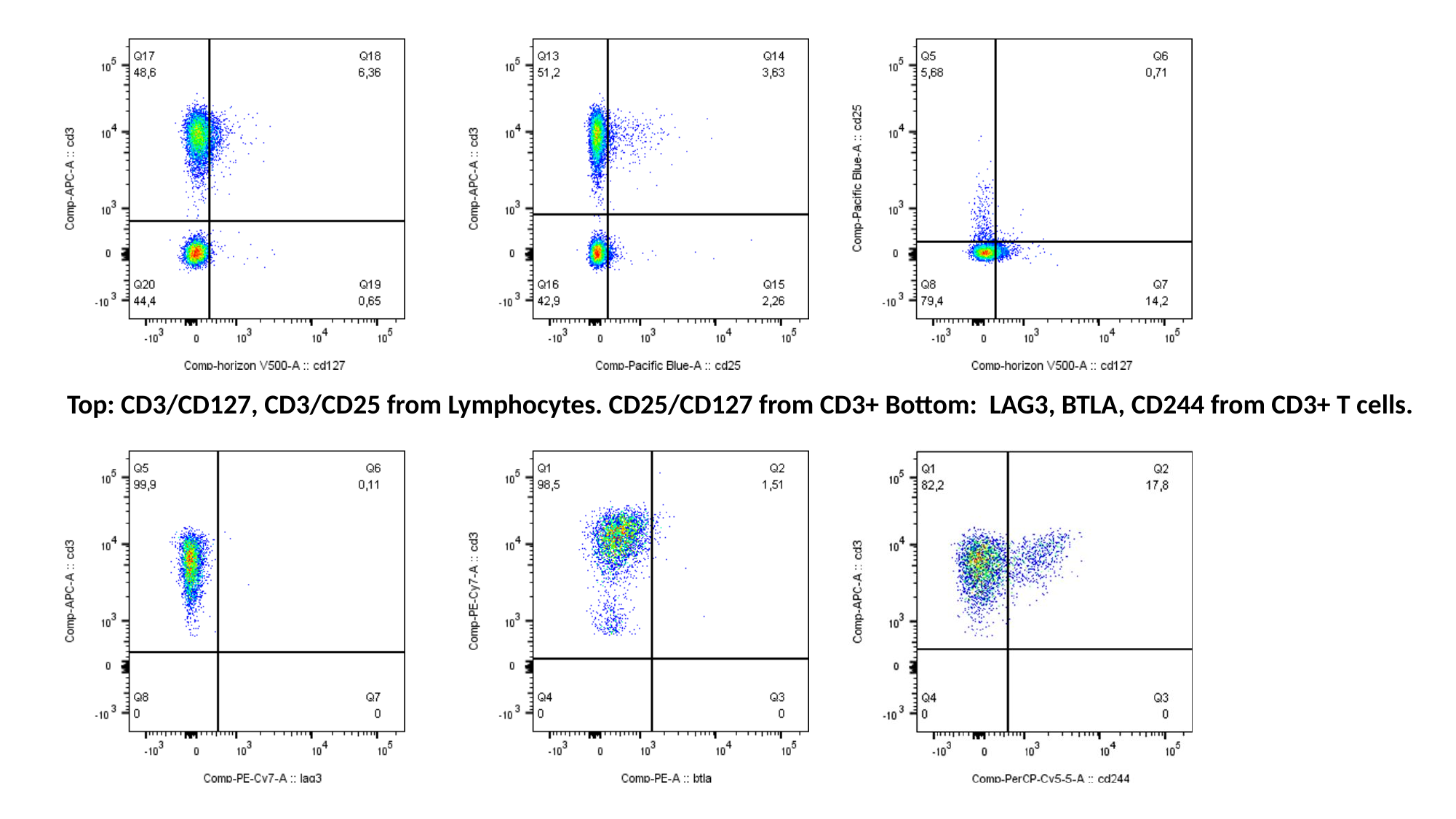

Top: CD3/CD127, CD3/CD25 from Lymphocytes. CD25/CD127 from CD3+ Bottom: LAG3, BTLA, CD244 from CD3+ T cells.

## Slide 9
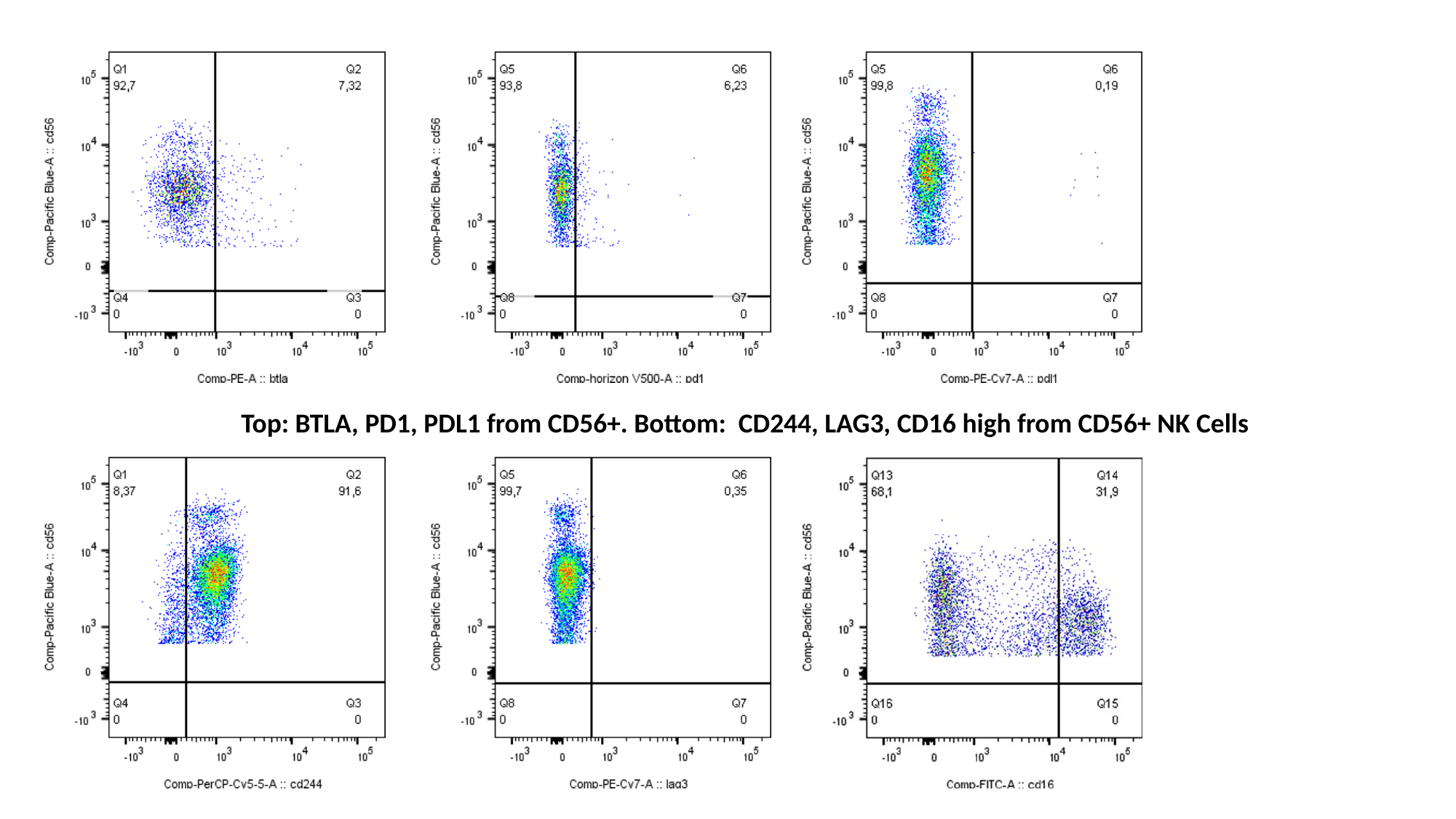

Top: BTLA, PD1, PDL1 from CD56+. Bottom: CD244, LAG3, CD16 high from CD56+ NK Cells
