# Supplementary material for: Immunometabolic Markers in a Small Patient Cohort Undergoing Immunotherapy
Source: Biomolecules. 2022 May 18;12(5):716. doi: 10.3390/biom12050716 (PMC9139165; doi:10.3390/biom12050716)

## Slide 1
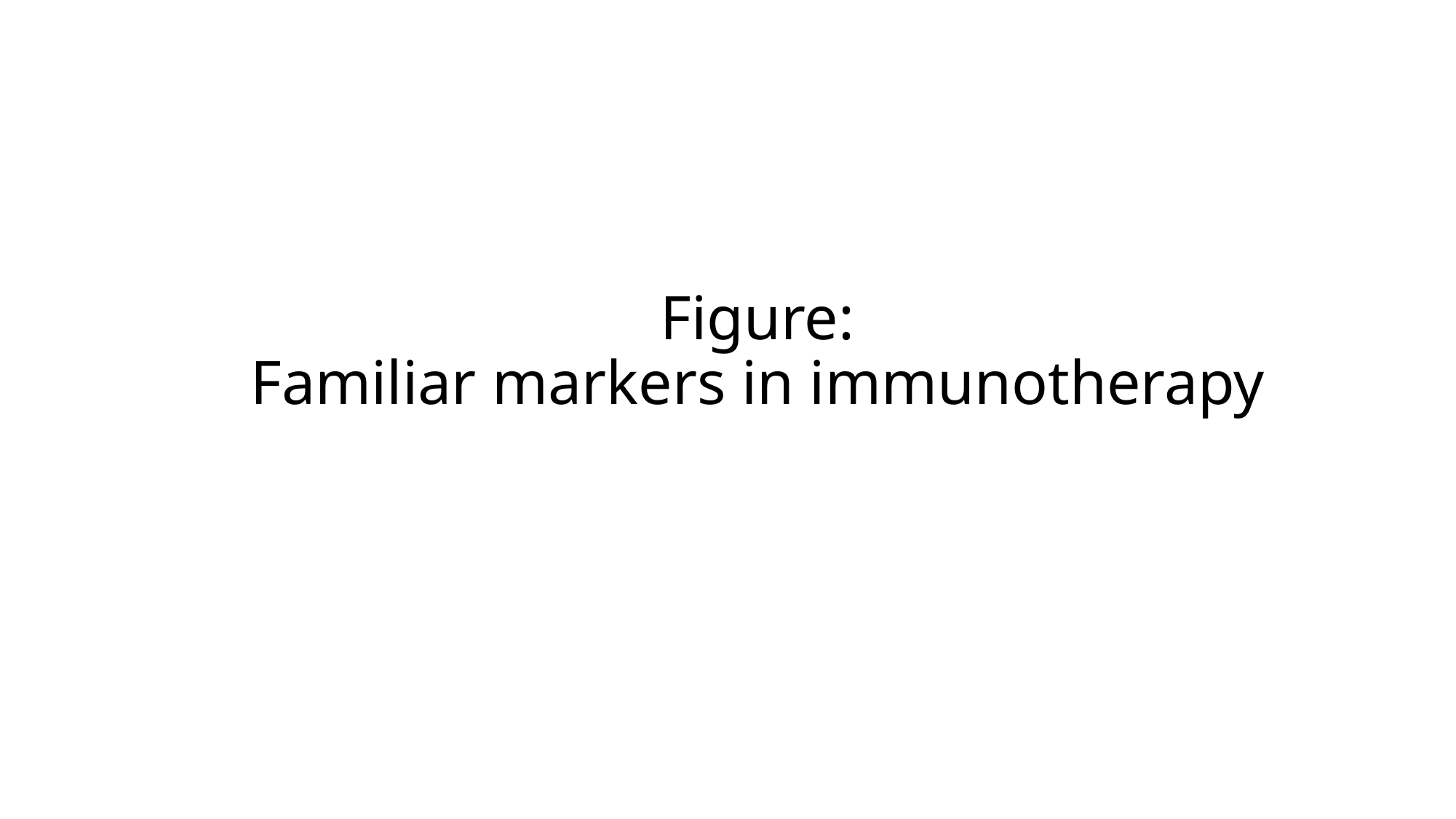

# Figure: Familiar markers in immunotherapy

## Slide 2
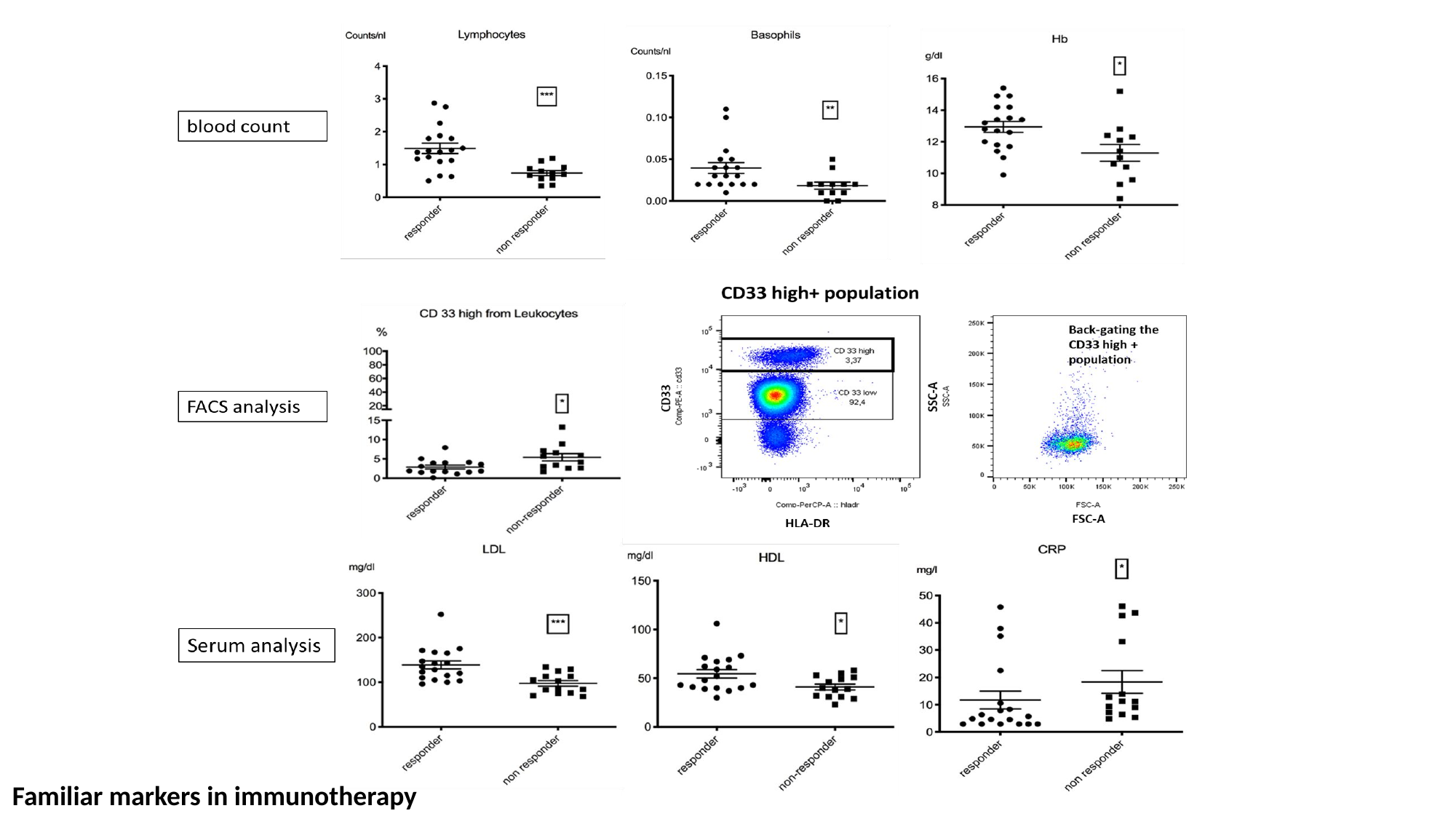

Familiar markers in immunotherapy

## Slide 3
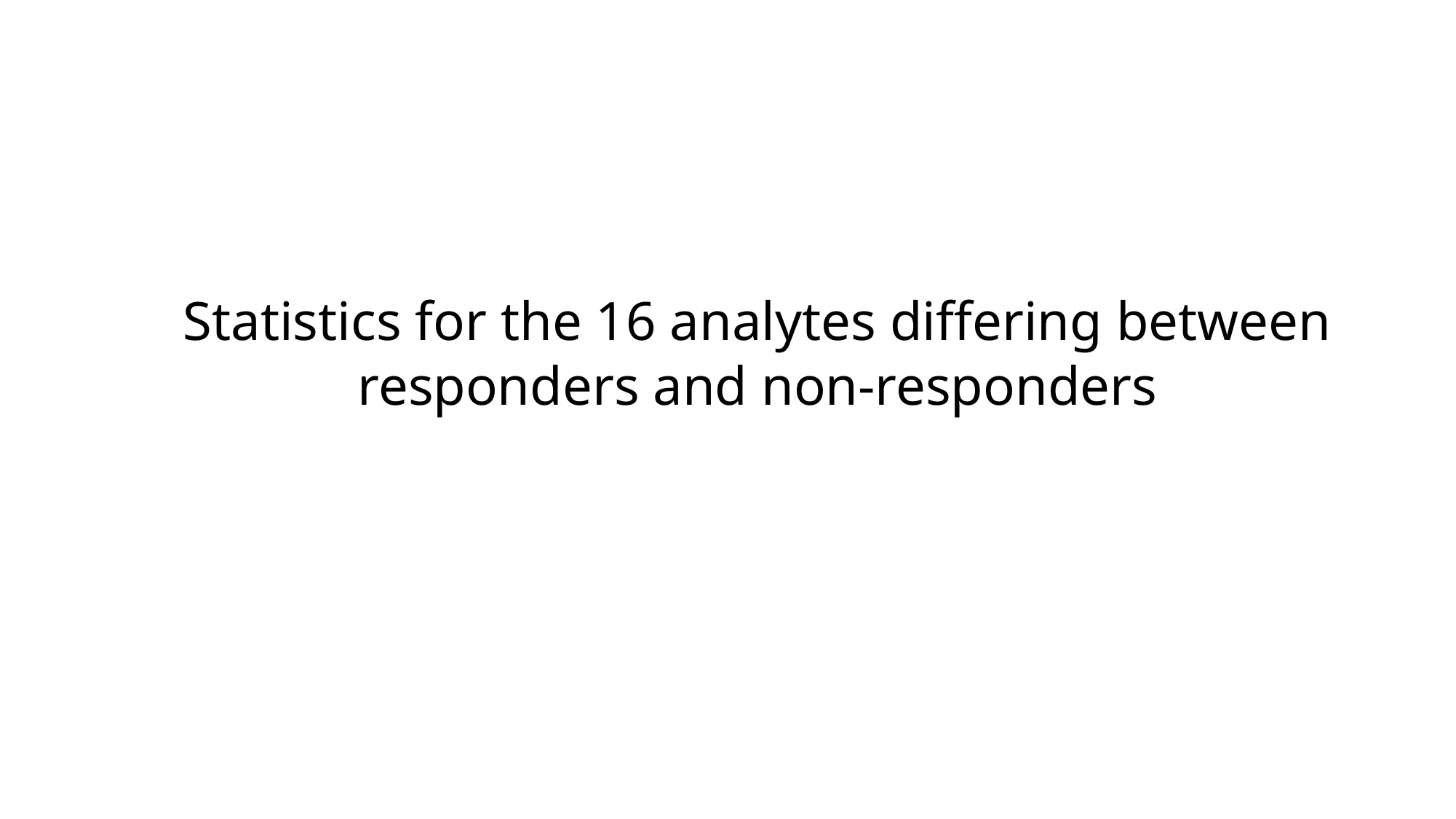

# Statistics for the 16 analytes differing between responders and non-responders

## Slide 4
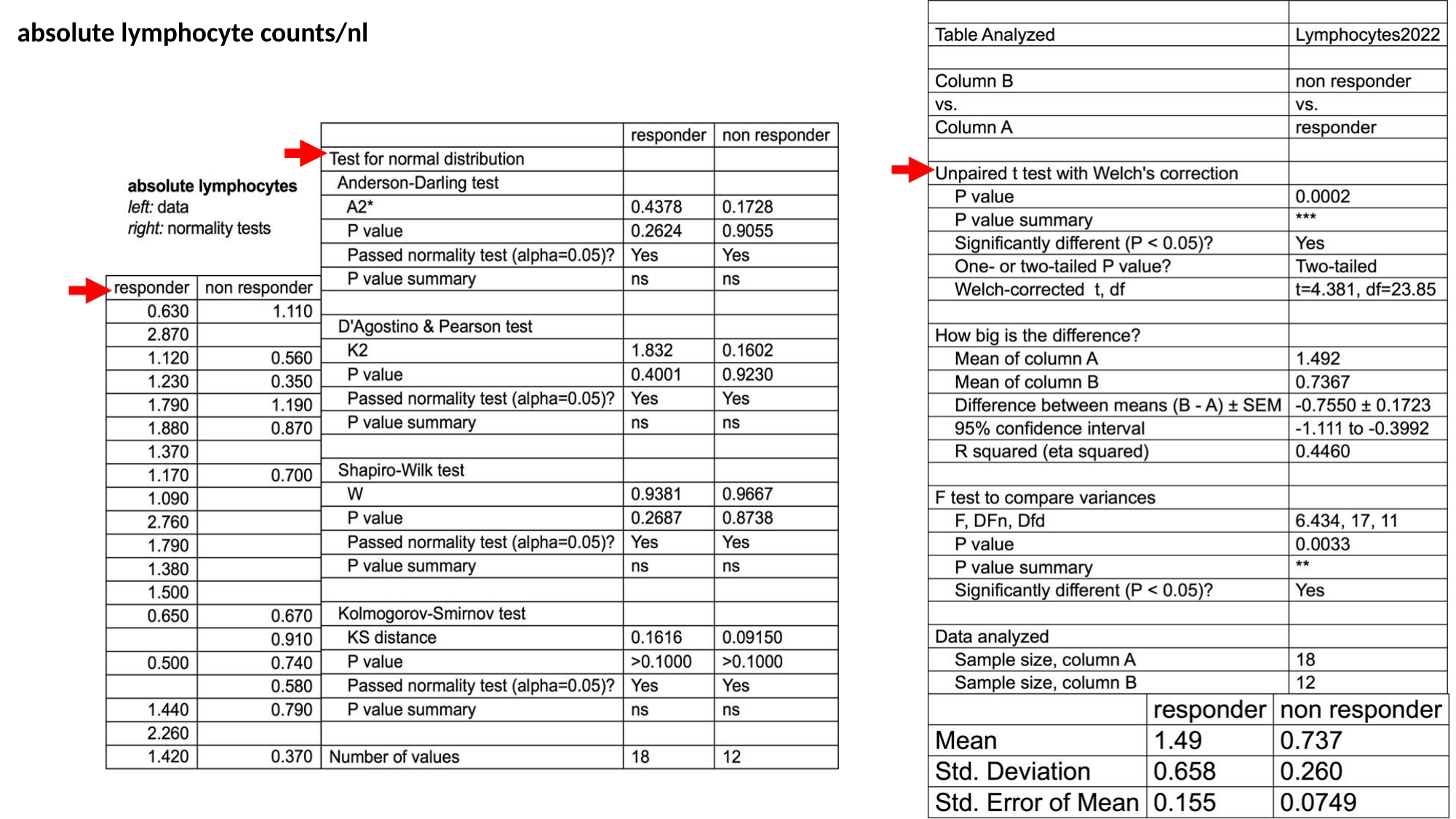

absolute lymphocyte counts/nl

## Slide 5
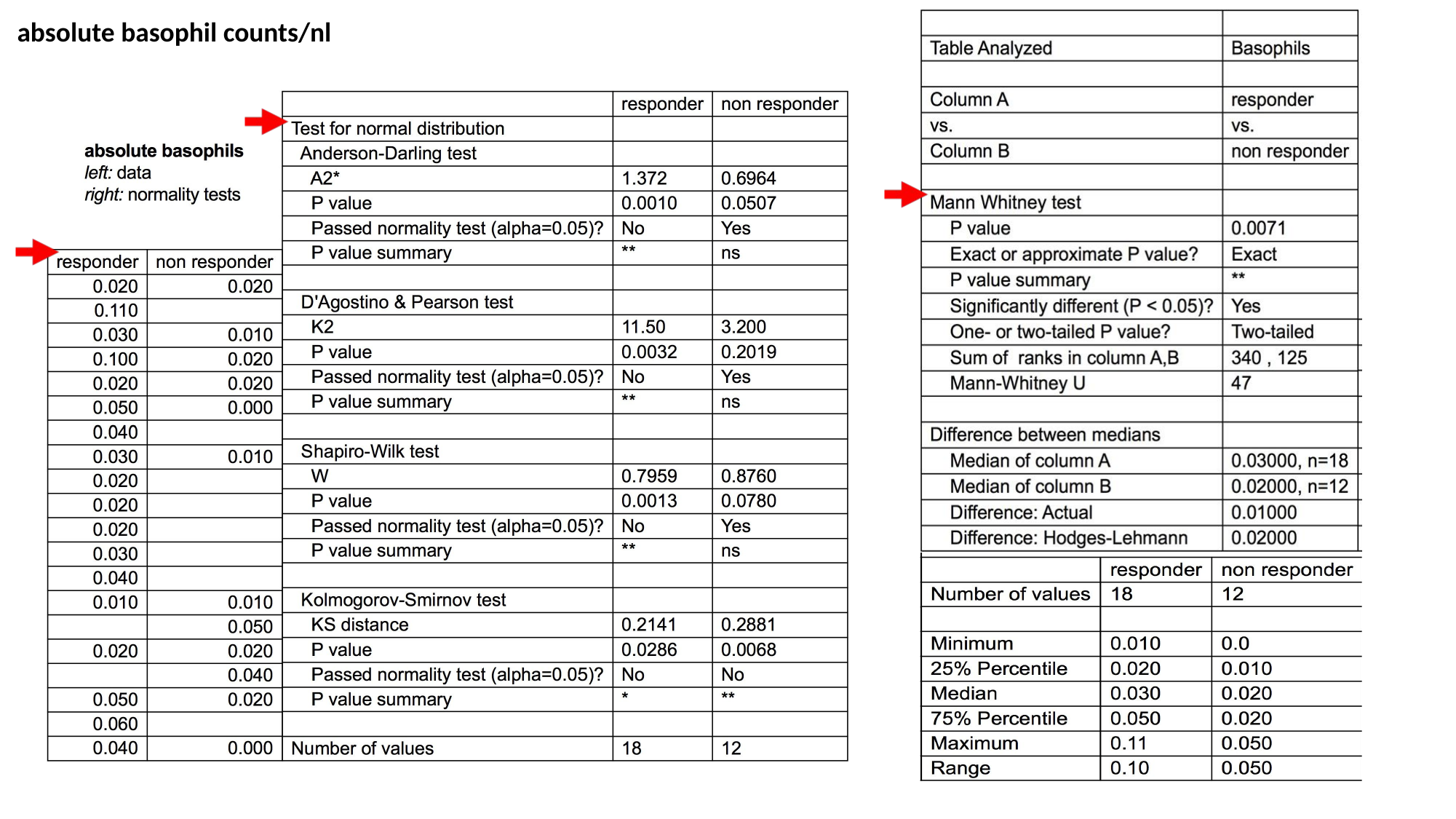

absolute basophil counts/nl

## Slide 6
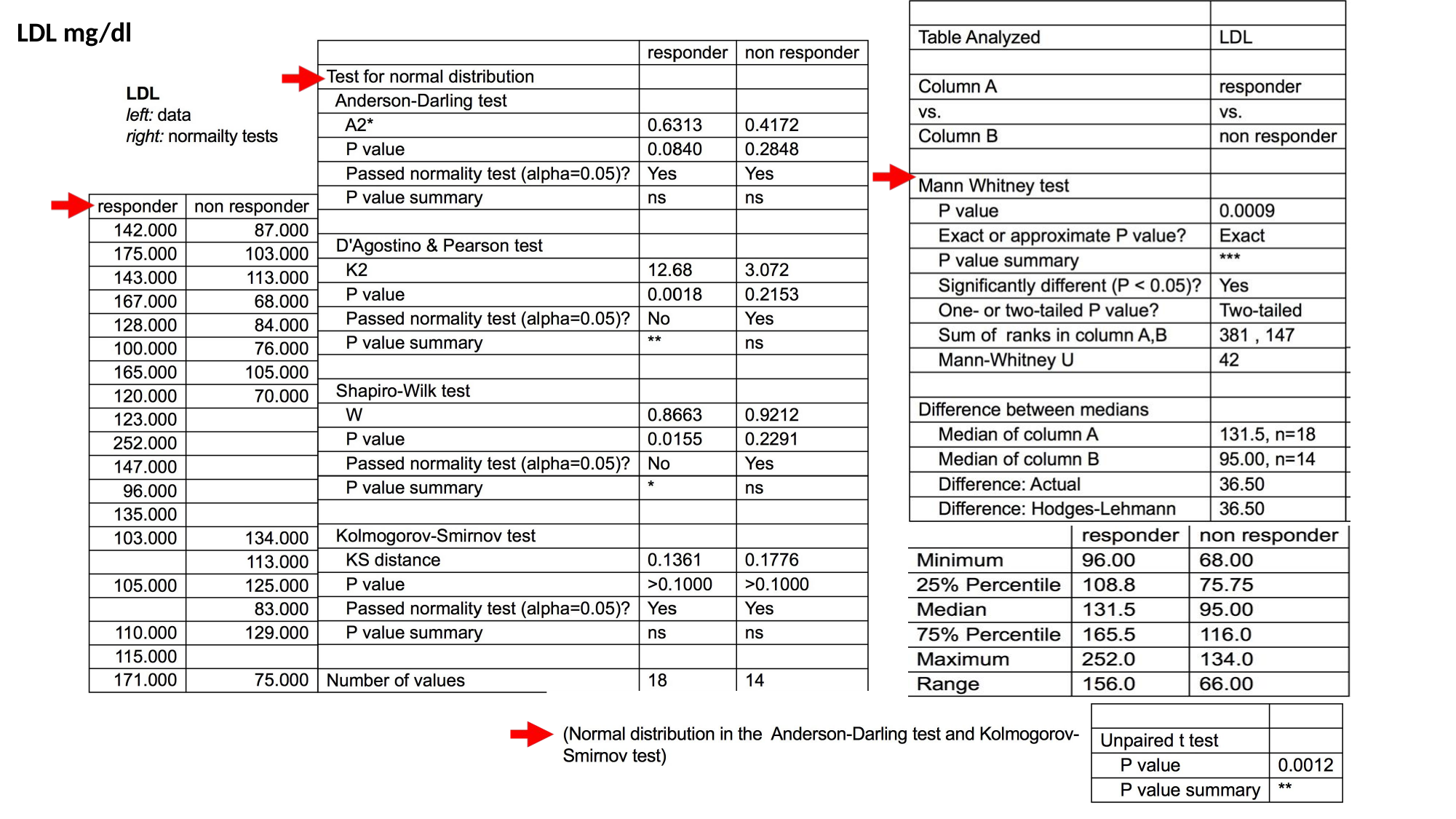

LDL mg/dl

## Slide 7
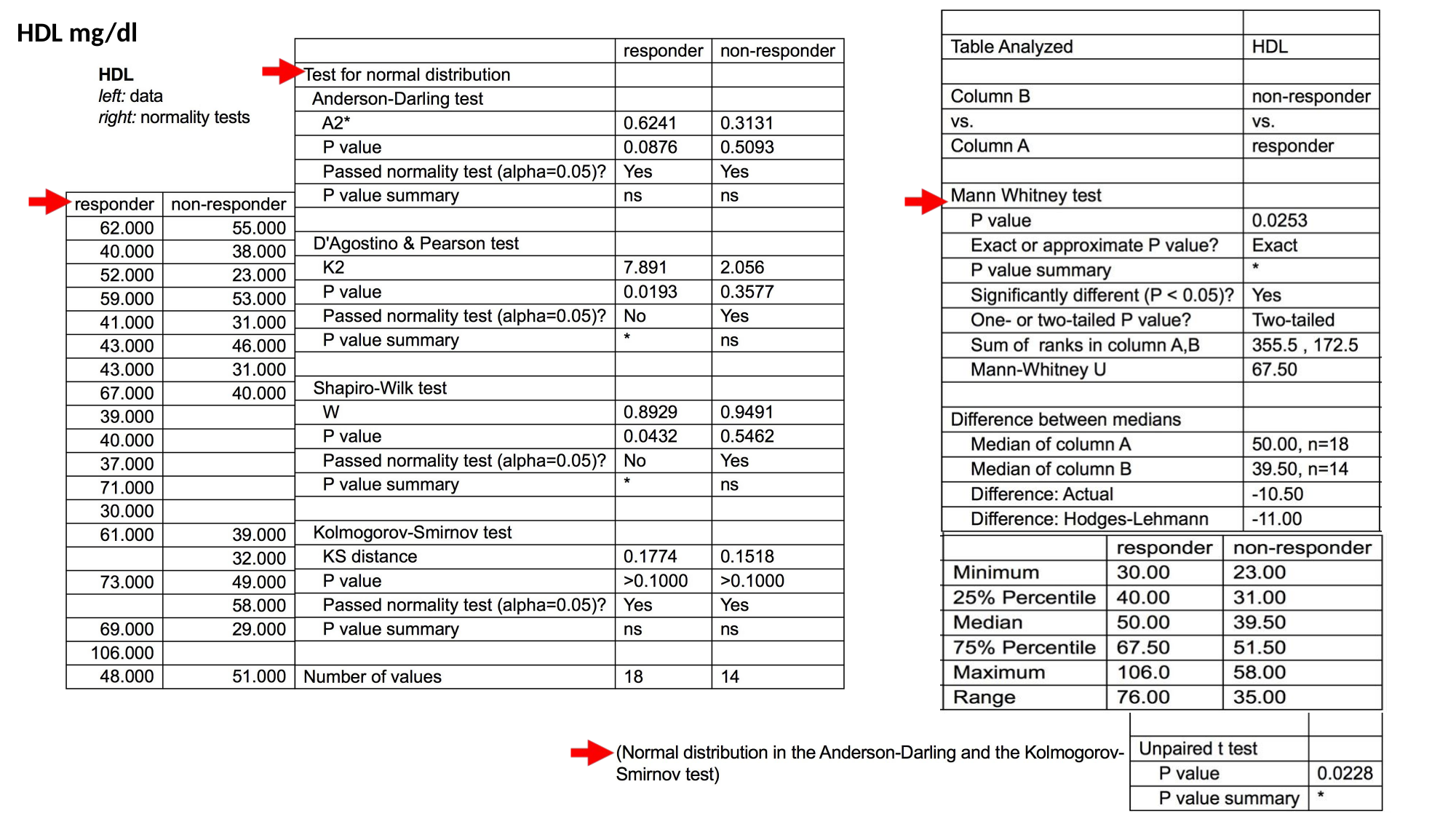

HDL mg/dl

## Slide 8
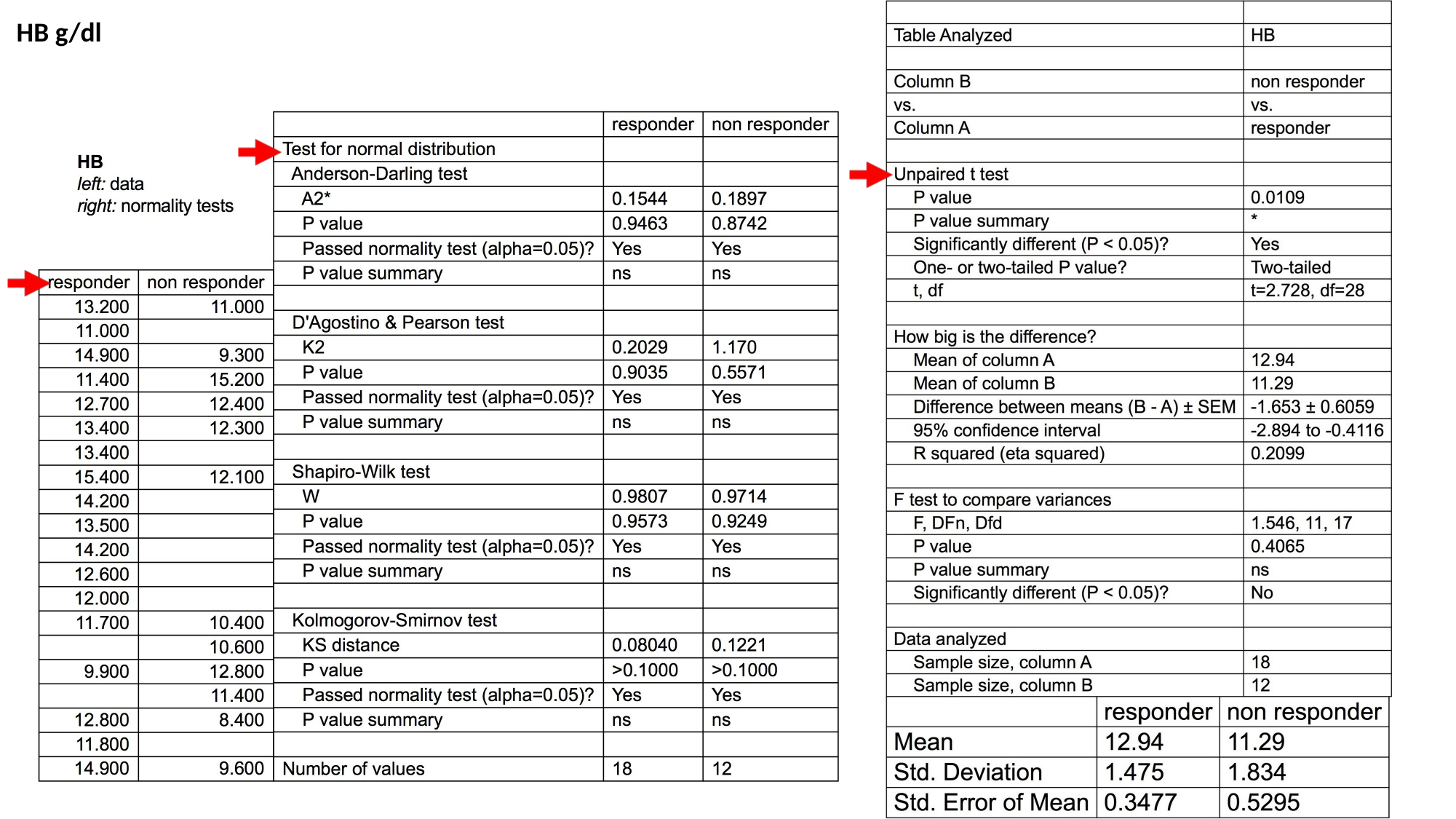

HB g/dl

## Slide 9
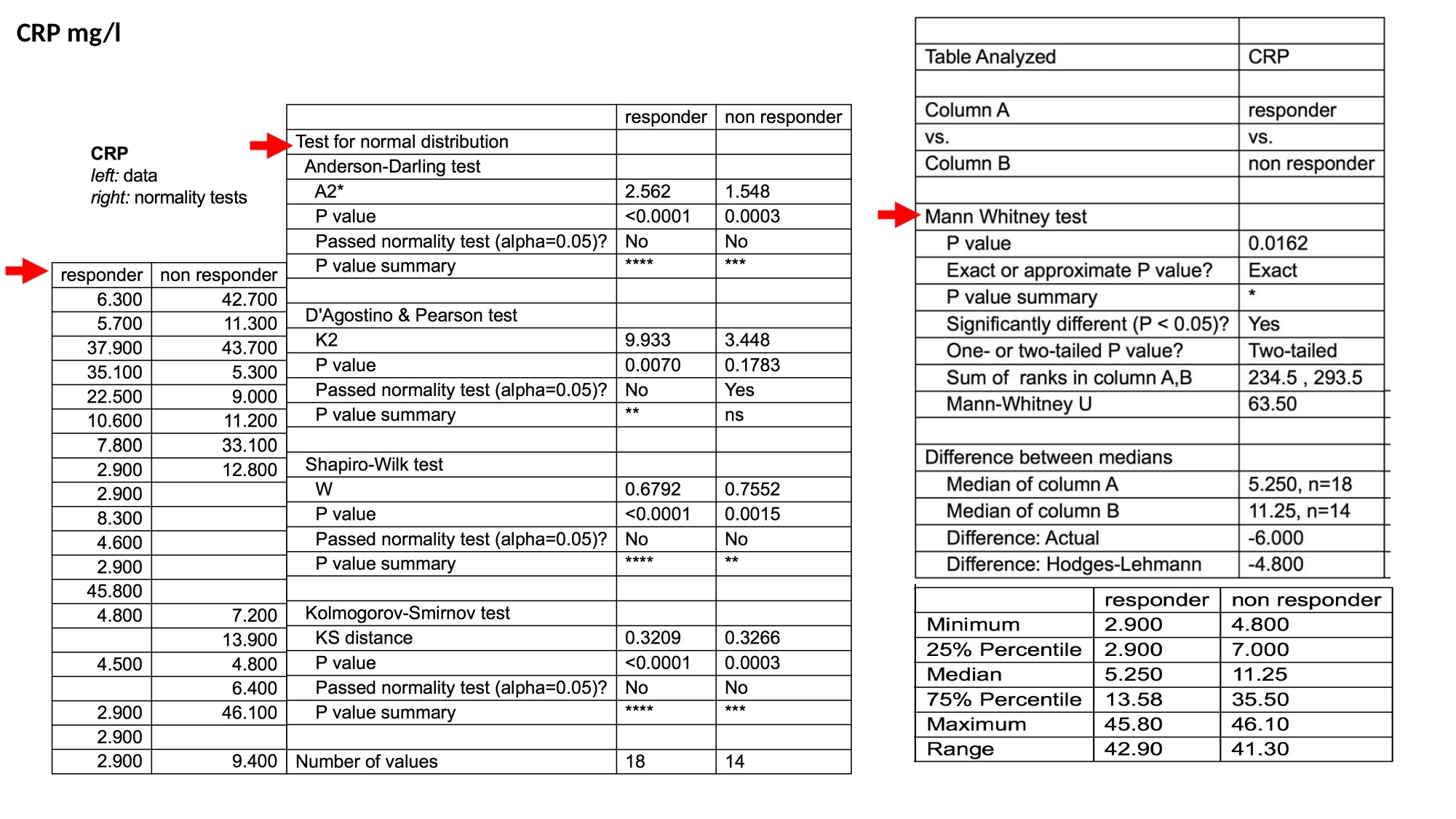

CRP mg/l

## Slide 10
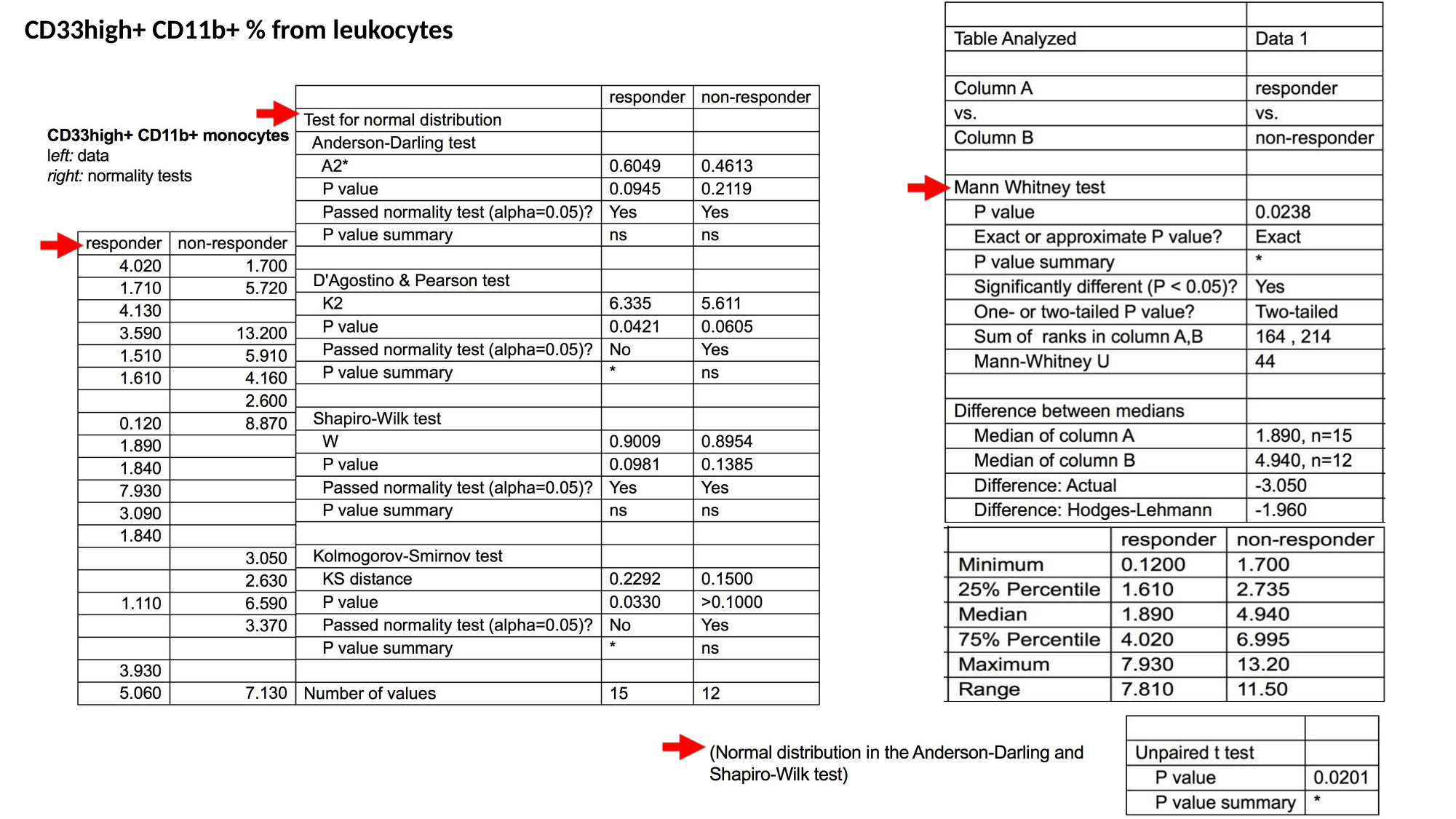

CD33high+ CD11b+ % from leukocytes

## Slide 11
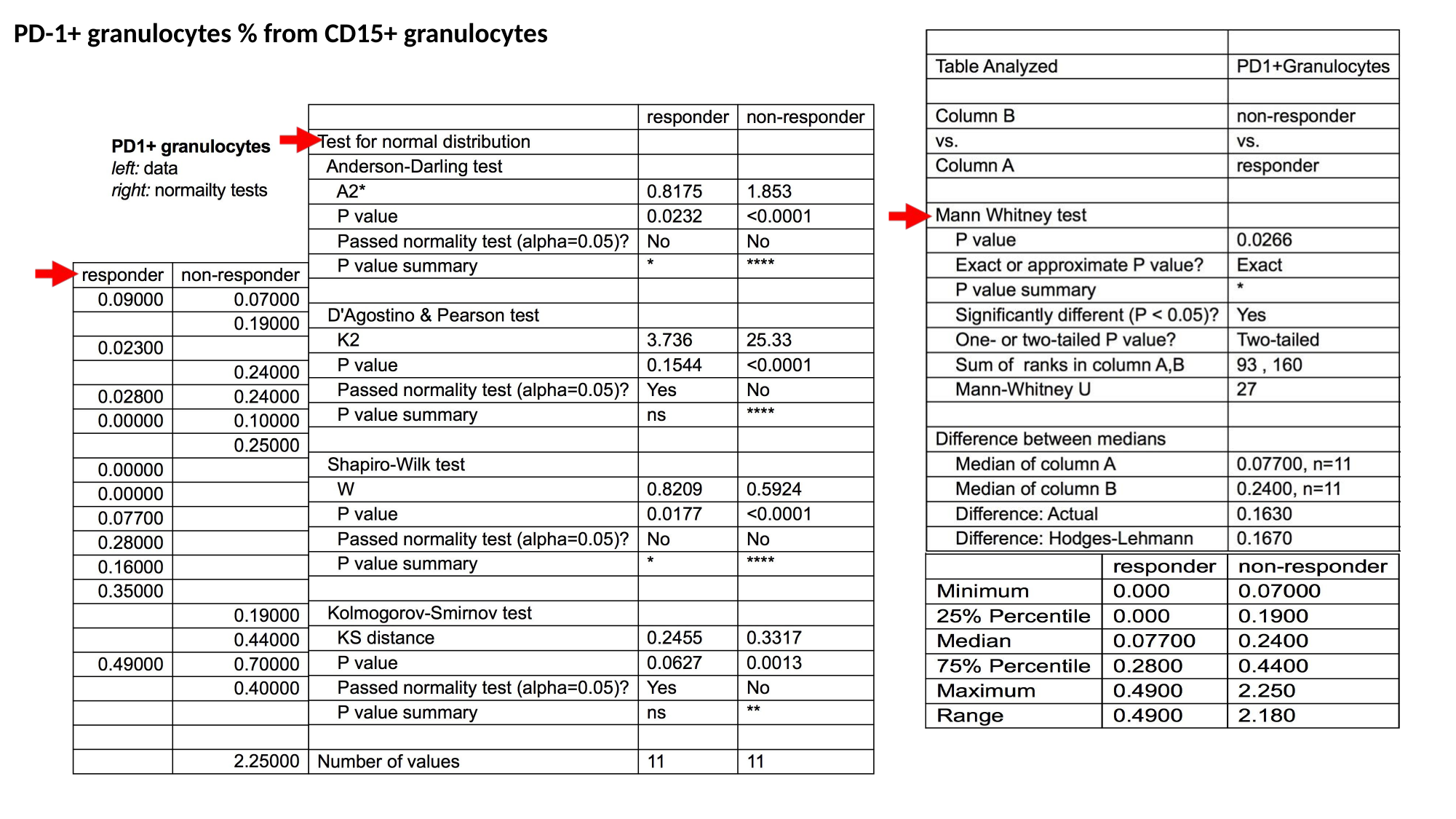

PD-1+ granulocytes % from CD15+ granulocytes

## Slide 12
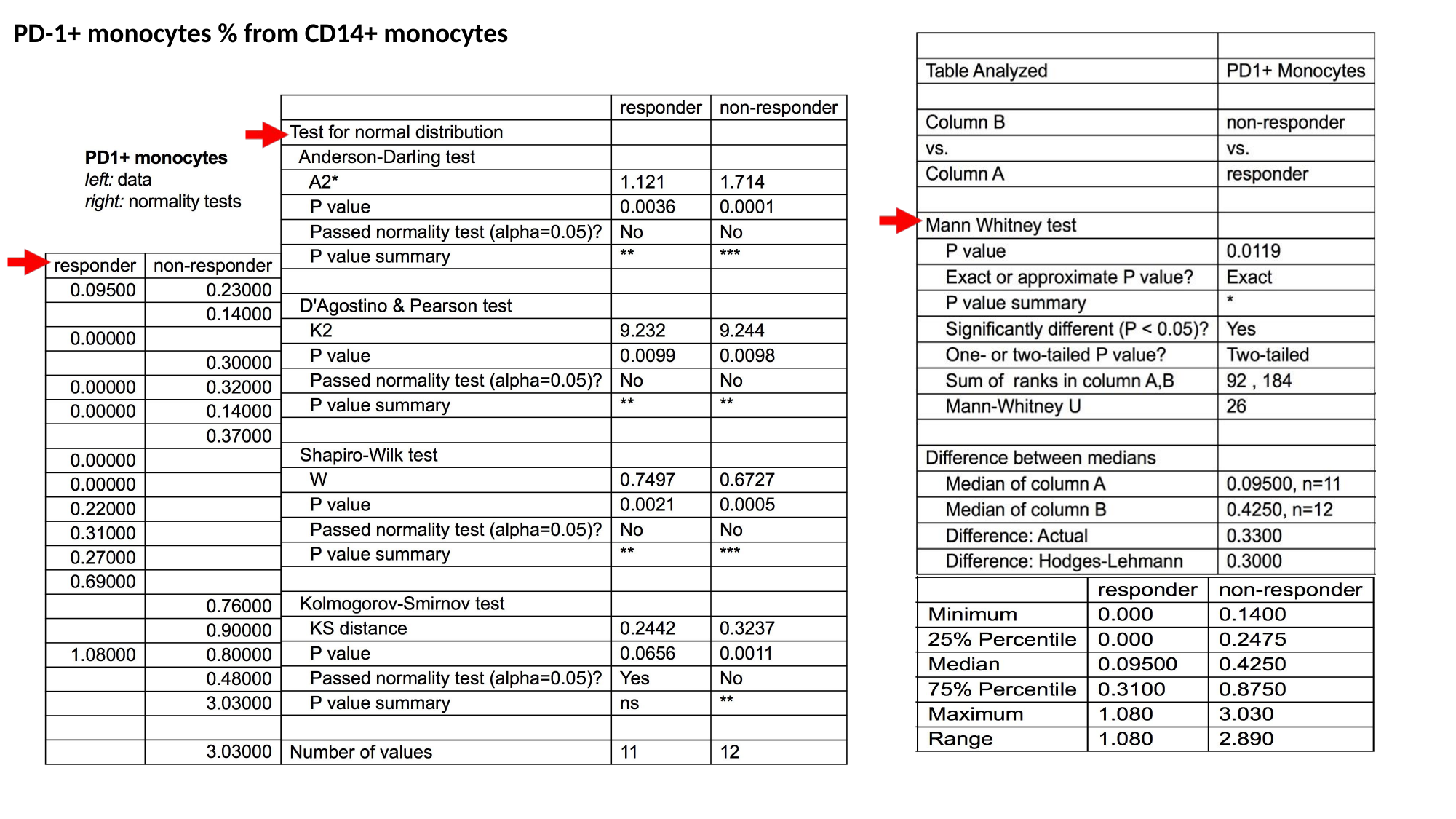

PD-1+ monocytes % from CD14+ monocytes

## Slide 13
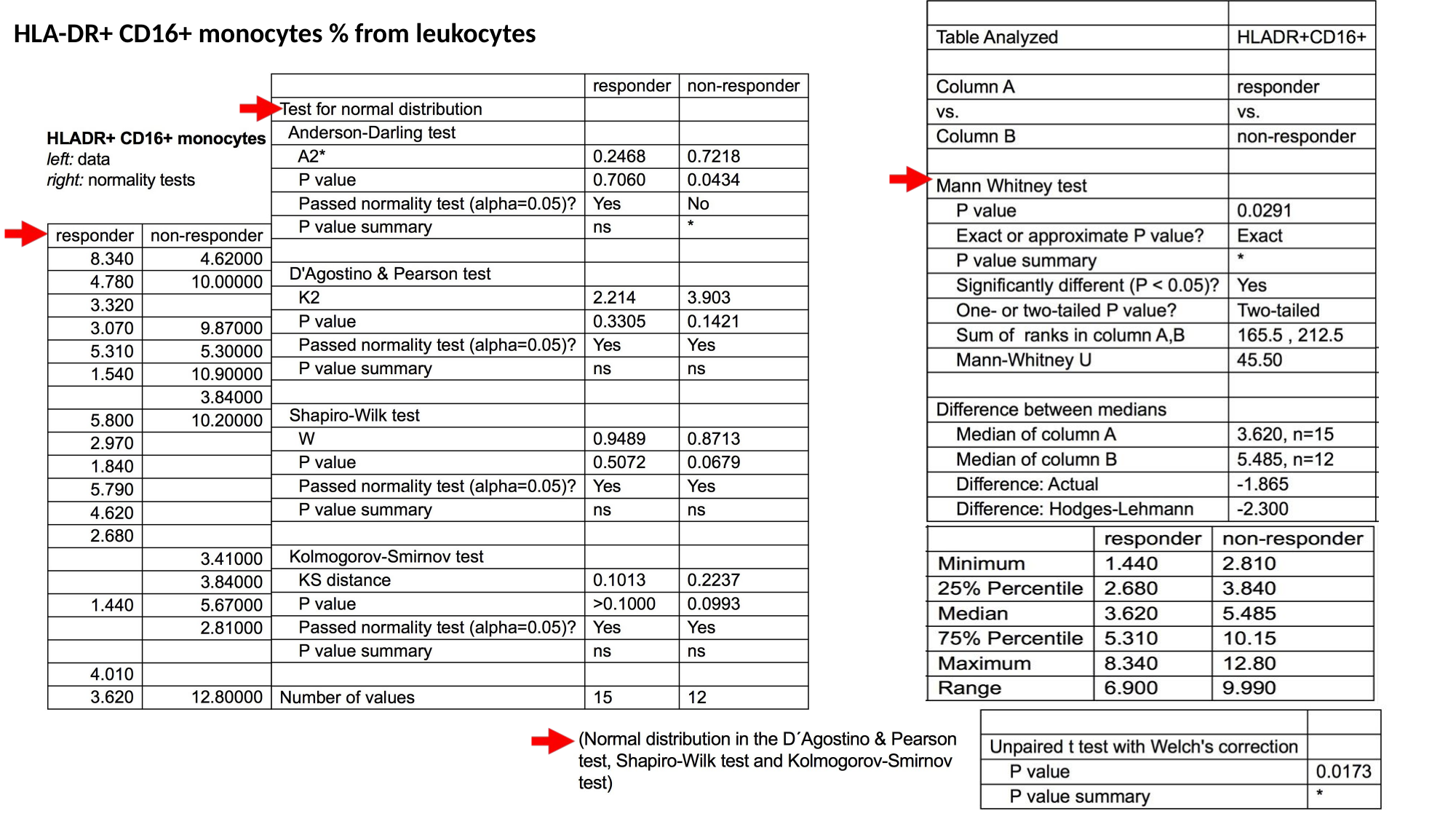

HLA-DR+ CD16+ monocytes % from leukocytes

## Slide 14
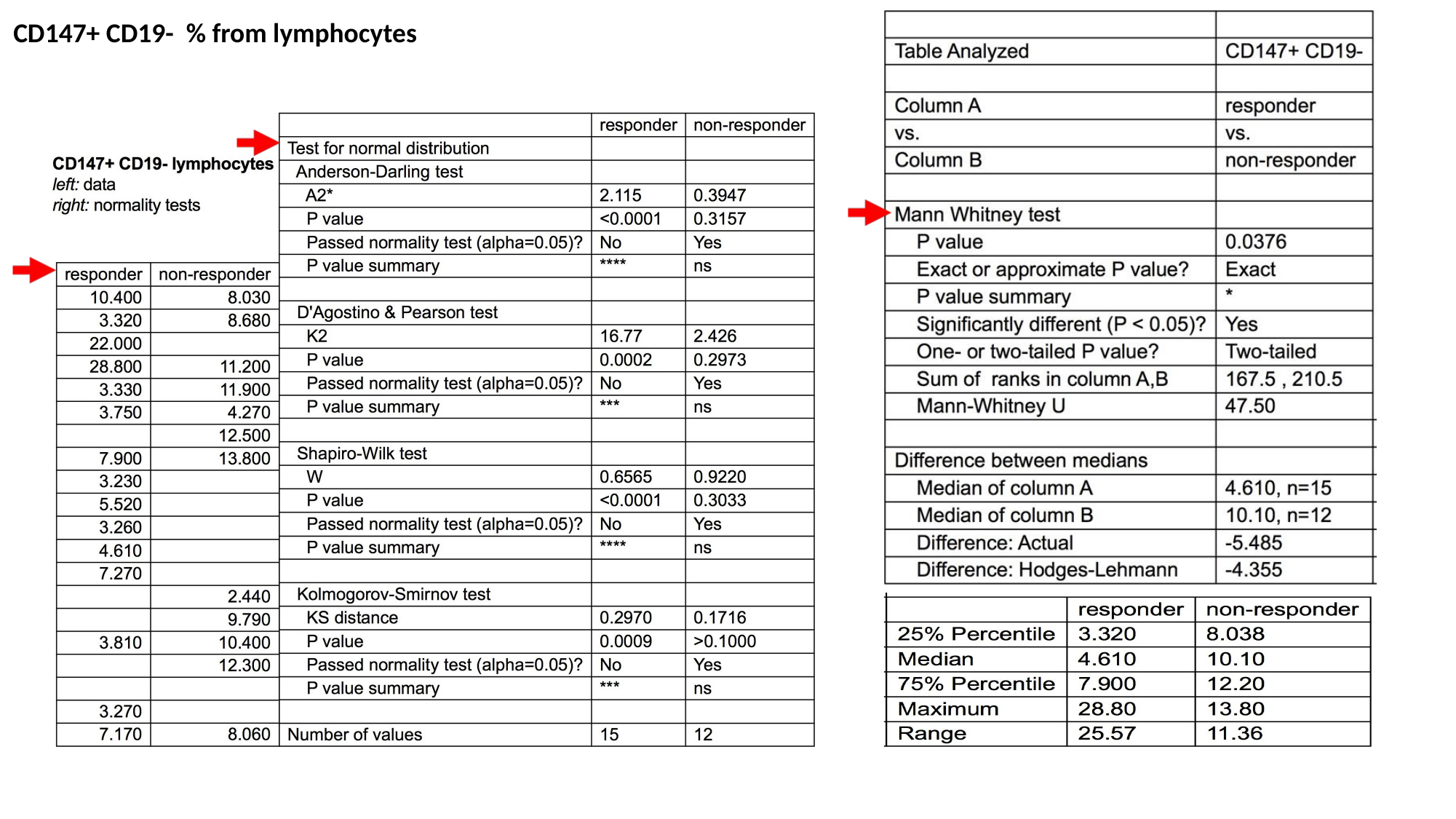

CD147+ CD19- % from lymphocytes

## Slide 15
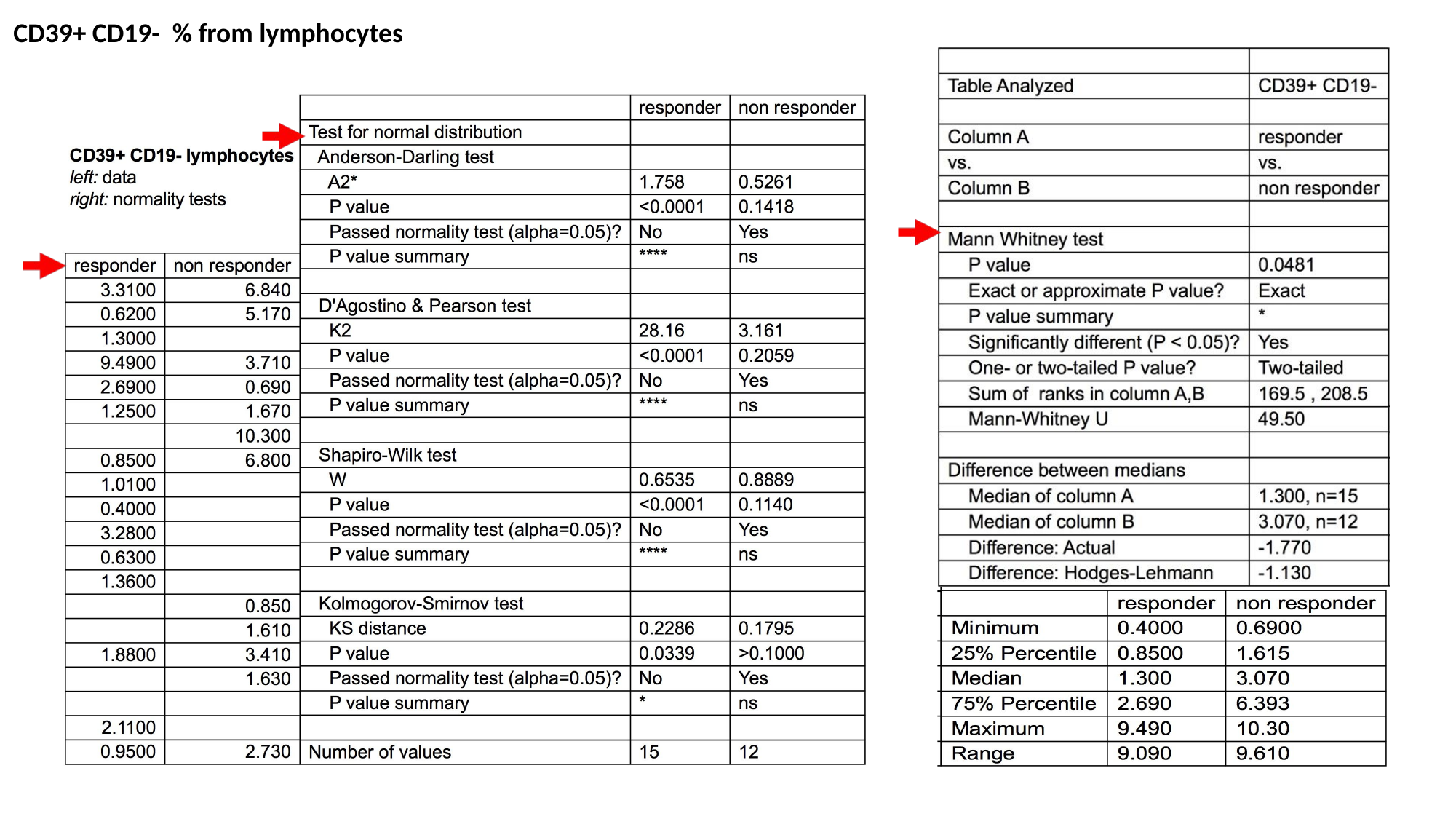

CD39+ CD19- % from lymphocytes

## Slide 16
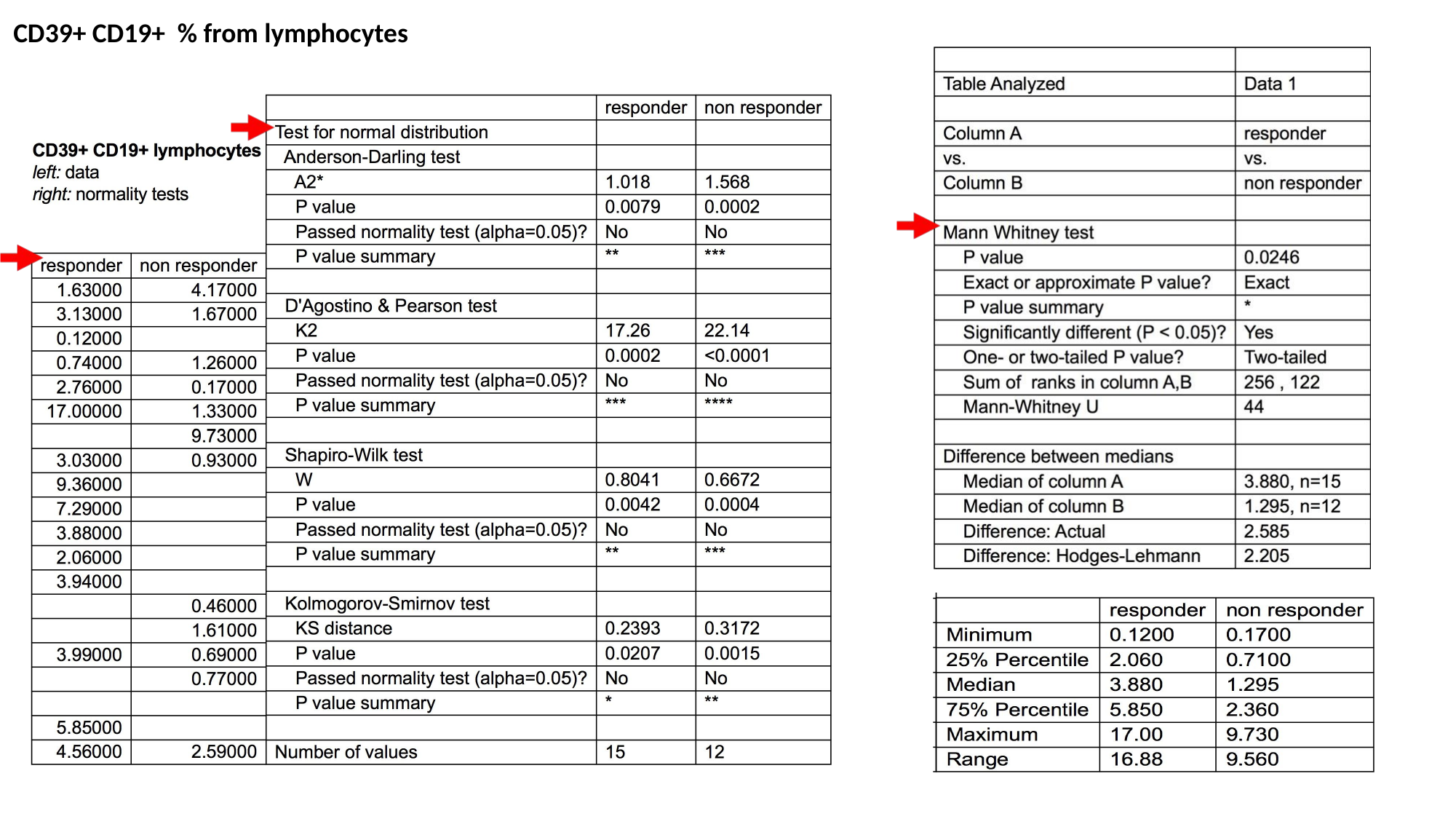

CD39+ CD19+ % from lymphocytes

## Slide 17
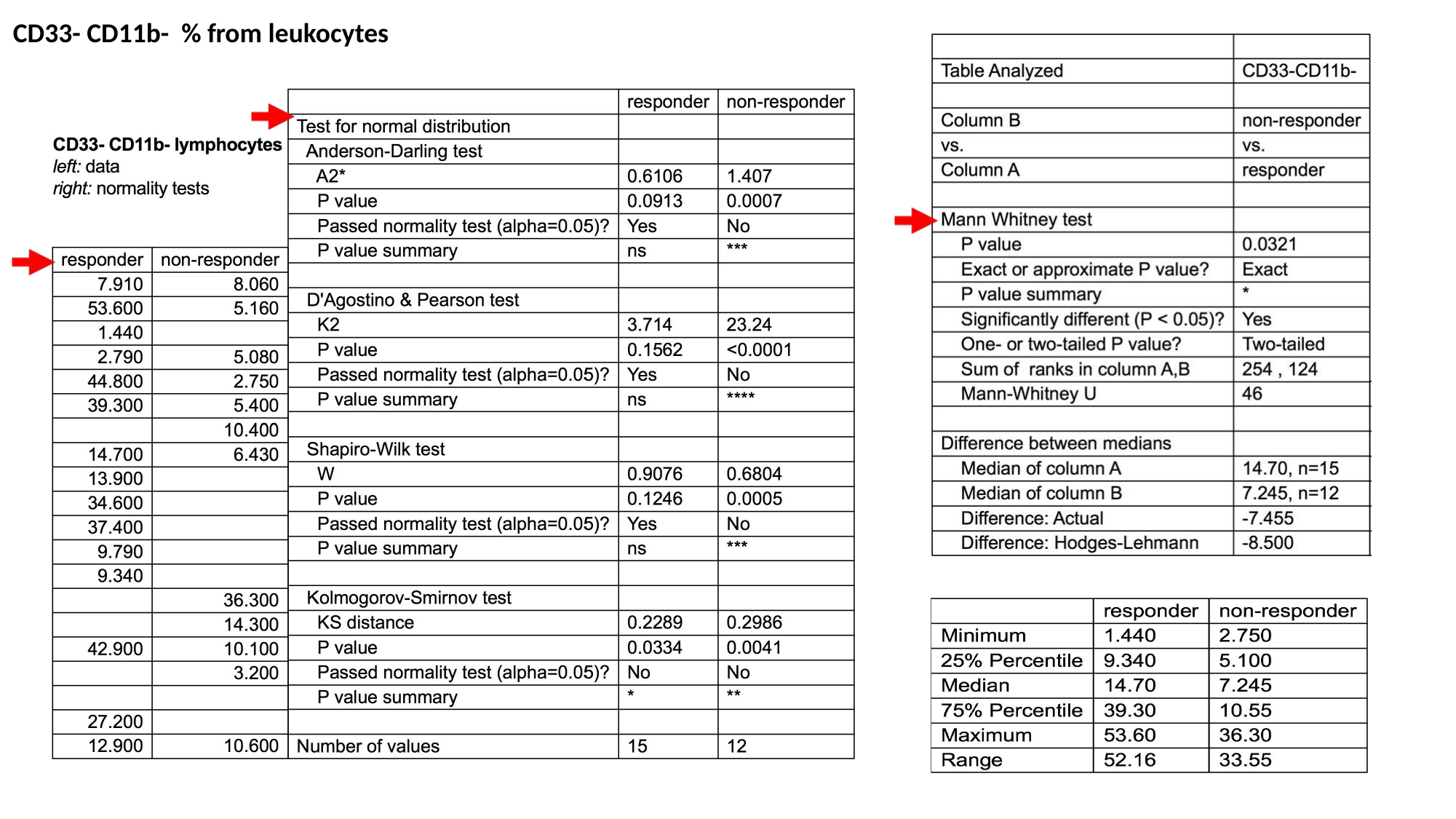

CD33- CD11b- % from leukocytes

## Slide 18
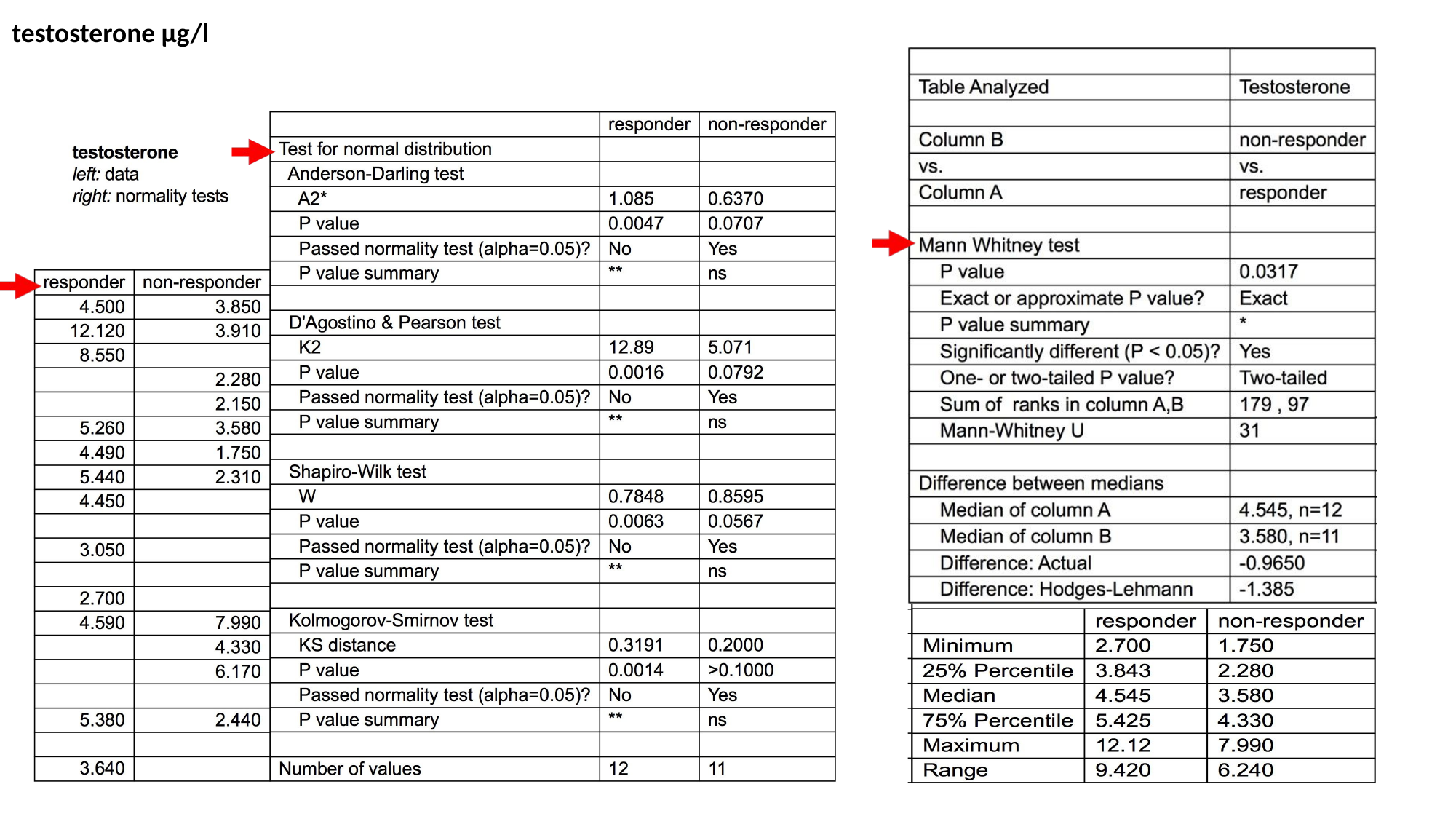

testosterone µg/l

## Slide 19
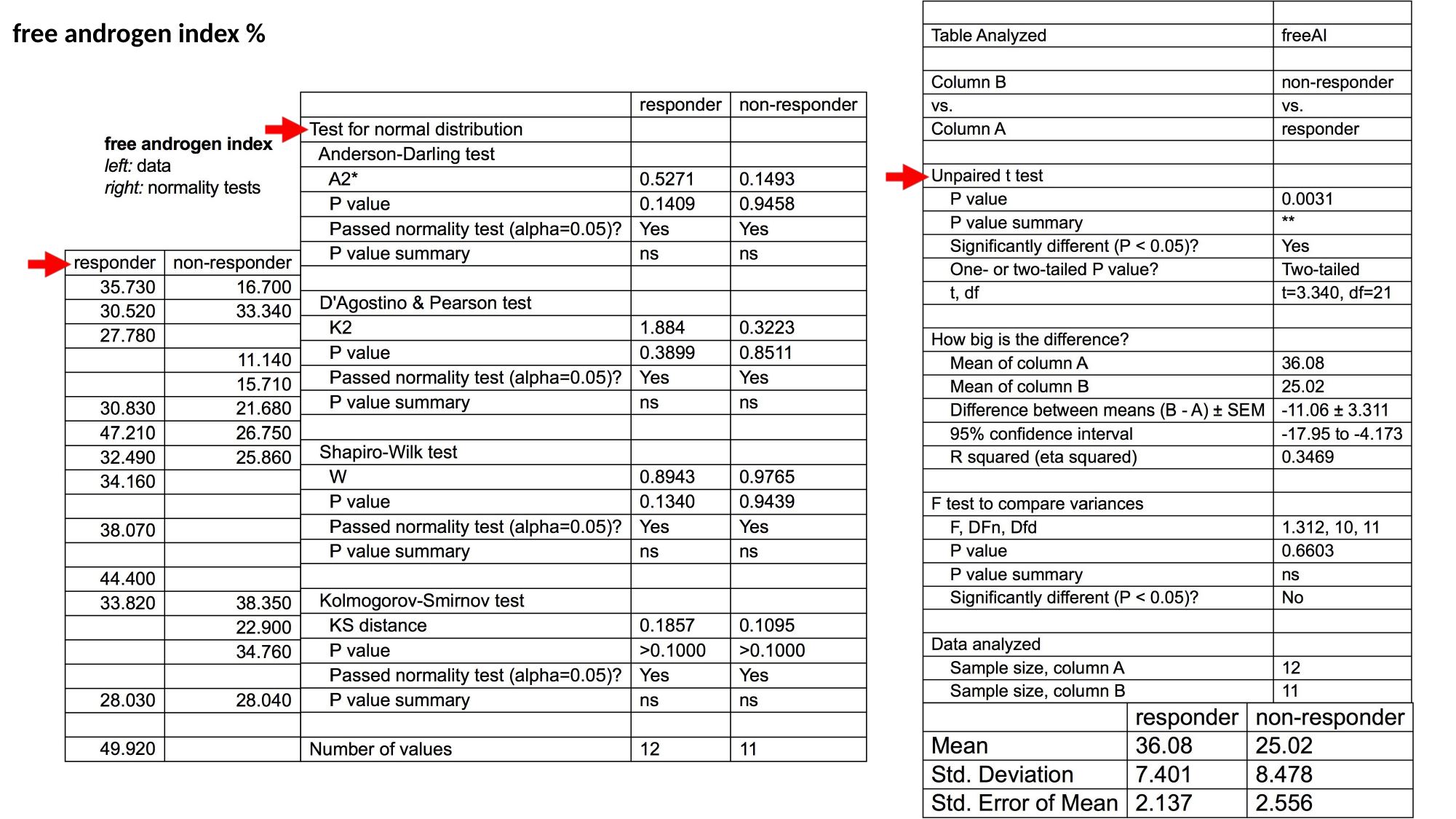

free androgen index %

## Slide 20
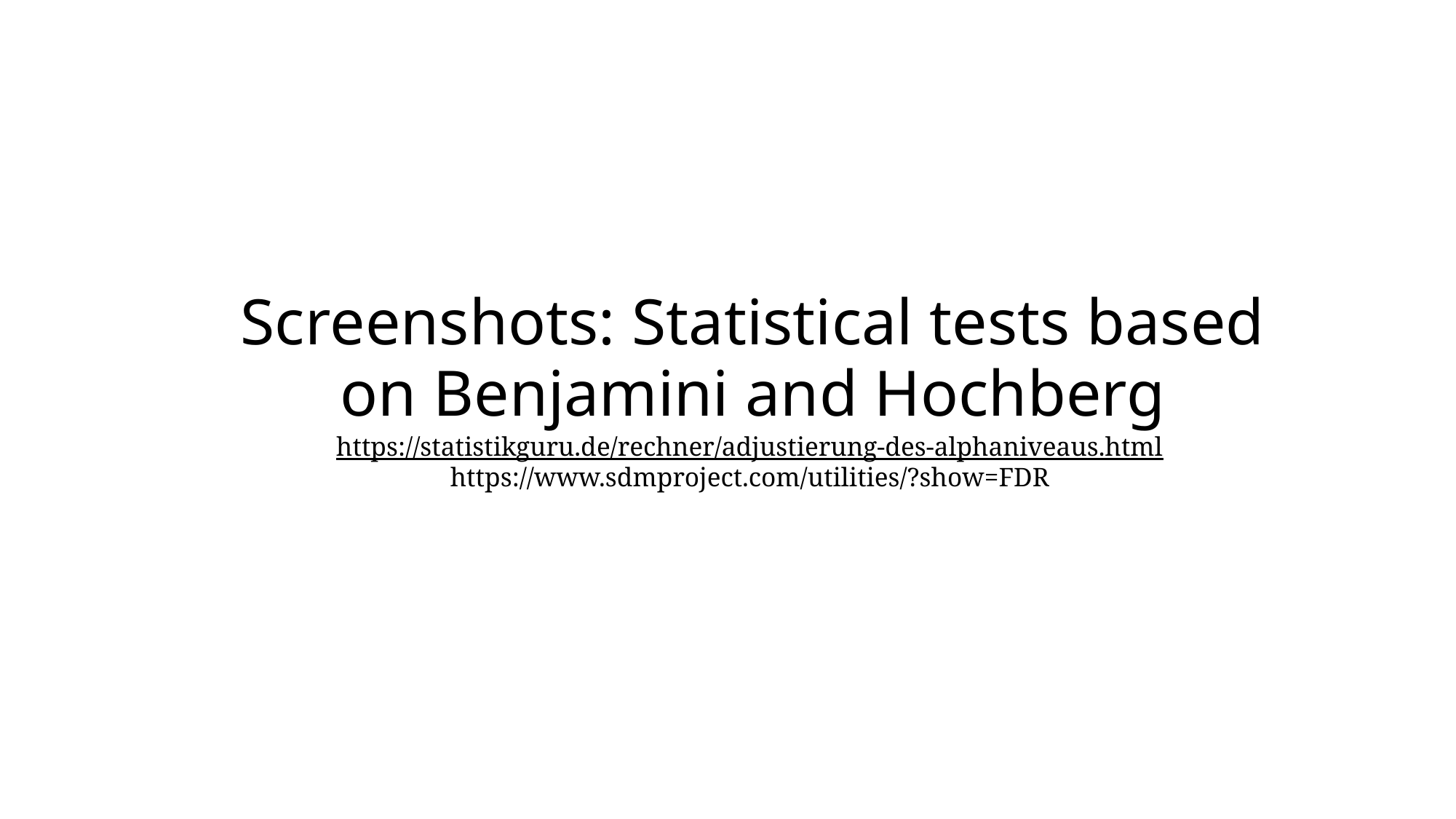

Screenshots: Statistical tests based on Benjamini and Hochberg
https://statistikguru.de/rechner/adjustierung-des-alphaniveaus.html https://www.sdmproject.com/utilities/?show=FDR

## Slide 21
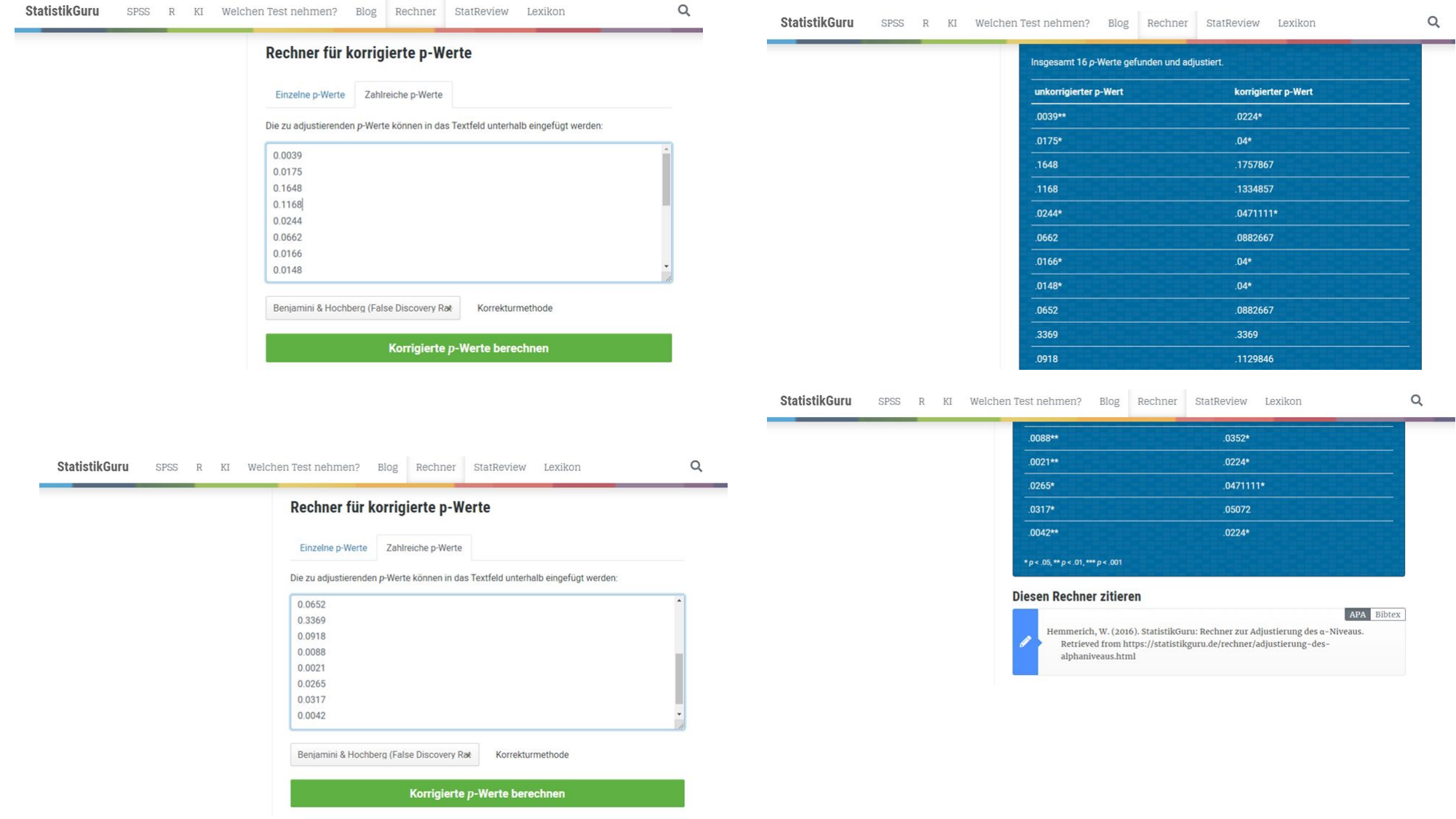

## Slide 22
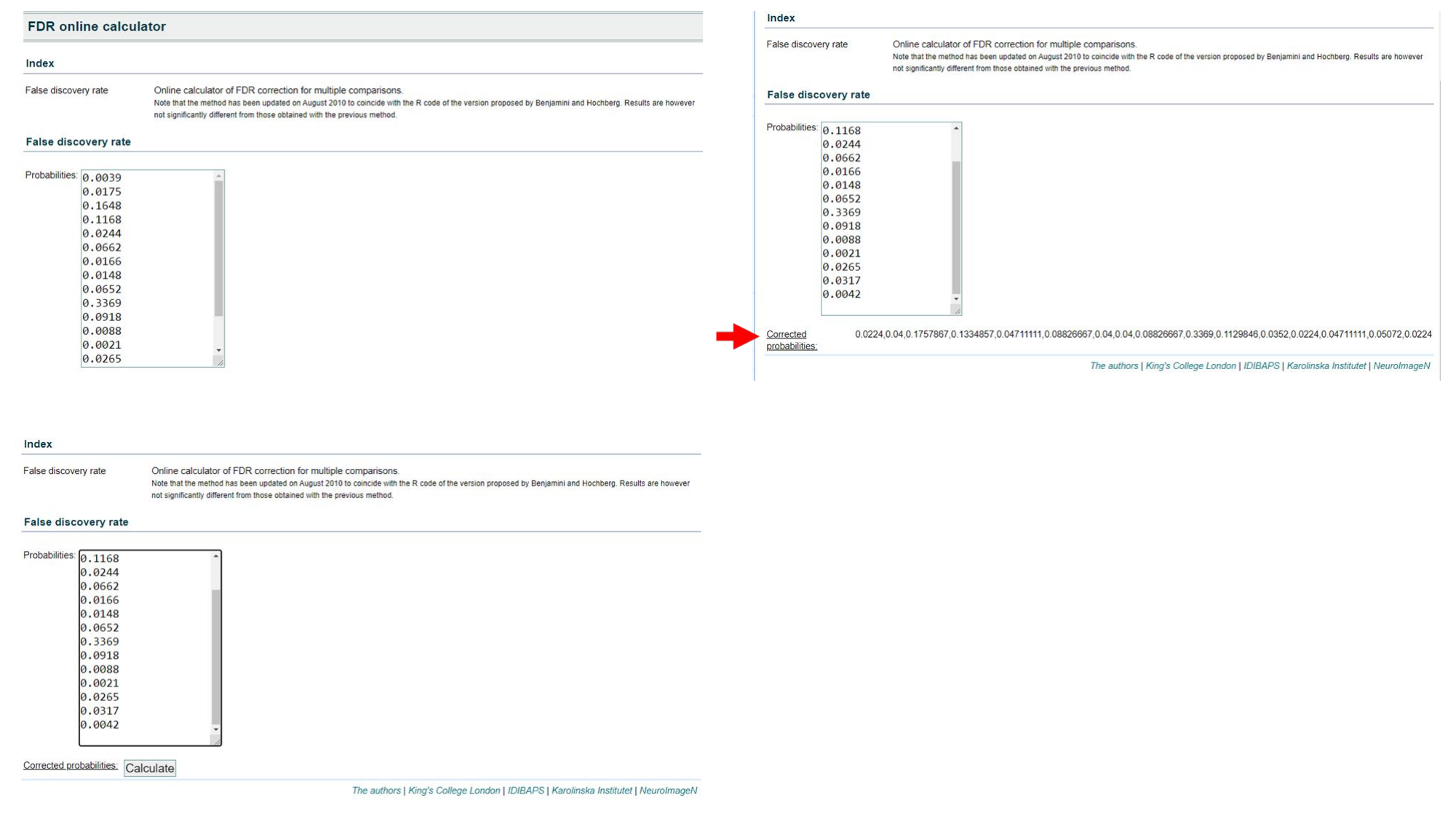

Supplement: Supplementary file 1 [file biomolecules-12-00716-s001.zip › Supplemental Data 2, Figure familiar markers and Statistics-Reports for the 16 markers differing between responders and non-responders.pptx]
