# Supplementary material for: Immunometabolic Markers in a Small Patient Cohort Undergoing Immunotherapy
Source: Biomolecules. 2022 May 18;12(5):716. doi: 10.3390/biom12050716 (PMC9139165; doi:10.3390/biom12050716)
Supplement: Supplementary file 1 [file biomolecules-12-00716-s001.zip › Supplemental Data 3, Tables (S3.1-S3.3).pdf]

Table Supplemental Data S3.1: Leukocytes and lymphocyte subsets. $p > 0.05$.

A

immune subset (absolute counts)	standard value	responder	non-responder
neutrophils	(1.56-6.13 /nl)	4.45/nl (+/- 1.2)	4.00/nl (+/- 2.50)
eosinophils	(0.04-0.36 /nl)	0.16/nl (+/- 0.09)	0.14/nl (+/- 0.11)
monocytes	(0.24-0.36 /nl)	0.56/nl (0.44/0.71)	0.5/nl (0.38/0.64)
platelets	(182-369 /nl)	274 /nl (+/- 76.6)	229.3 /nl (+/- 114.5)

B

immune subset	% from population	responder	non-responder
granulocytes	CD15 from Leukocytes	60.39 % (+/- 23.4)	73.34 % (+/- 13.2)
monocytes	CD14 from Leukocytes	3.19 % (1.96/5.08)	6.21 % (2.99/9.03)
lymphocytes	FSC/SSC from Leukocytes	21.95 % (9.59/41.23)	9.53 % (5.81/12.40)
T cells	CD3 from Lymphocytes	67.91 % (+/- 13.04)	63.99 % (+/- 11.91)
B cells	CD19 from Lymphocytes	6.91 % (+/- 5.11)	4.823 % (+/- 2.73)
CD123+BDCA2+pDC	pDC from lymphocytes	0.04 % (0.02/0.06)	0.04 % (0.02/0.06)
NK cells	CD56 from Lymphocytes	21.66 % (+/- 9.99)	25.53 % (+/- 12.25)

C

T cell subsets	% from population	responder	non-responder
PD-1+	% from T Cells	12.4 % (3.07/23.3)	8.46 % (5.31/14.6)
PD-L1+	% from T Cells	0.16 % (0.11/0.49)	0.34 % (0.11/0.46)
LAG-3+	% from T Cells	0.07 % (+/- 0.03)	0.16 % (+/- 0.14)
BTLA+	% from T Cells	54.72 % (+/- 23.2)	44.81 % (+/- 19)
CD244+	% from T Cells	17.80 % (10/34.5)	11.35 % (5.91/31.7)
TIM-3+	% from T Cells	0.46 % (0.29/0.96)	0.56 % (0.16/0.9)
CD3+/CD4+	% from Lymphocytes	47.06 % (+/- 13.37)	37.93 % (+/- 13.84)
CD3+/CD8+	% from Lymphocytes	13.3 % (9.84/27.68)	20.06 % (13.4/30.53)
CD3+/CD69+	% from Lymphocytes	1.01 % (0.24/1.45)	1.04 % (0.73/2.04)
CD3+/CD25+	% from Lymphocytes	2.76 % (+/- 1.55)	3.68 % (+/- 2.76)
CD3+/CD127+	% from Lymphocytes	30.36 % (+/- 16.44)	16.22 % (+/- 14.68)
CD25+/CD127-	% from T Cells	2.29 % (+/- 1.81)	4.8 % (+/- 3.7)

D

B cell subsets	% from population	responder	non-responder
PD-1+	% from B Cells	0.38 % (0/1.78)	1.57 % (0.52/1.92)
PD-L1+	% from B Cells	3.07 % (+/- 1.41)	4.24 % (+/- 2.13)
LAG-3+	% from B Cells	0.17 % (0.13/0.4)	0.3 % (0.04/1.62)
BTLA+	% from B Cells	99.7 % (99.2/99.9)	99.4 % (97.3/99.6)
CD244+	% from B Cells	1.55 % (0.72/2.96)	1.59 % (0.66/3.29)
CD27+/CD24+	% from B Cells	8.71 % (2.96/17.2)	7.31 % (1.68/13.03)
CD27+/CD38+	% from B Cells	2.22 % (1.23/3.89)	2.64 % (1.77/6.97)
IgD+	% from B Cells	84.9 % (77.1/89.3)	83.3 % (53.85/90.38)
IgM+	% from B Cells	41.1 % (+/- 26.35)	34.33 % (+/- 20.92)
IgD+/IgM+	% from B Cells	37.76 % (+/- 24.33)	30.52 % (+/- 19.28)

E

NK cell subsets	% from population	responder	non-responder
PD-1+	% from NK Cells	0.82 % (0/4.02)	1.65 % (1.34/2.13)
PD-L1+	% from NK Cells	0.52 % (0.24/0.64)	0.46 % (0.31/2.23)
LAG-3+	% from NK Cells	0.35 % (0.18/0.49)	0.5 % (0.31/0.82)
BTLA+	% from NK Cells	5.66 % (4/13.95)	3.58 % (2.45/9.26)
CD244+	% from NK Cells	94.4 % (90.4/97.6)	93.8 % (84.63/95.38)
CD56 high+	% from NK Cells	4.56 % (2.79/10.5)	6.57 % (3.69/13.16)
CD3+/CD56+	% from Lymphocytes	3.66 % (1.8/7.42)	2.00 % (0.98/6.96)
CD16+	% from NK Cells	58.7 % (39.2/84.0)	73.05 % (37.28/84.35)

A Routine laboratory leukocyte subsets and platelets. **B** Relative leukocyte and lymphocyte subsets acquired by flow cytometry. **C** T cell subsets, activation markers and checkpoint markers. **D** B cell subsets and checkpoint markers. **E** NK cell subsets and checkpoint markers. Depending on normality tests the mean with standard deviation (+/-) or median with (25%/75%) percentile is shown.

Supplemental Data S3.2: Myeloid subsets. $p > 0.05$.

A

myeloid cell subsets	% from population	responder	non-responder
PD-L1+	% from granulocytes	0.05 % (0.02/0.09)	0.06 % (0.02/0.12)
LAG-3+	% from granulocytes	0.02 % (0.01/0.03)	0.02 % (0.01/0.05)
BTLA+	% from granulocytes	0.1 % (0.03/0.38)	0.09 % (0.04/0.23)
CD244+	% from granulocytes	0.77 % (0.27/0.88)	0.73 % (0.26/1.15)
HLA-DR+	% from granulocytes	0.55 % (0.21/0.91)	0.26 % (0.10/0.89)

PD-L1+	% from monocytes	0.48 % (0.3/1.8)	0.37 % (0.15/1.16)
LAG-3	% from monocytes	0.27 % (0.05/1.49)	0.24 % (0.08/0.48)
BTLA	% from monocytes	0.52 % (0.13/2.58)	0.35 % (0.10/1.42)
HLA-DR-	% from monocytes	1.73 % (0.71/10)	3.22 % (1.32/7.92)

B

myeloid markers and CD16	% from population	responder	non-responder
CD33+ CD11b+	% from Leukocytes	56.9 % (+/- 31.83)	76.53 % (+/- 16.25)
CD33+ CD11b-	% from Leukocytes	0.44 % (0.16/0.78)	0.31 % (0.16/0.99)
CD33- CD11b+	% from Leukocytes	8.53 % (2.49/24.4)	6.49 % (2.21/29.65)
HLA-DR+ CD16++	% from Leukocytes	0.84 % (+/- 0.63)	0.99 % (+/- 0.56)
HLA-DR+ CD16-	% from Leukocytes	4.21 % (1.67/8.76)	3.98 % (1.05/5.09)

A Checkpoint markers on myeloid cell subsets. **B** Populations with the myeloid markers CD33/CD11b and HLA-DR/CD16 were analysed considering the whole Leukocyte population. Depending on normality tests the mean with standard deviation (+/-) or median with (25%/75%) percentile is shown.

Supplemental Data S3.3: Metabolic and hormonal markers. $p > 0.05$.

metabolic markers	standard value	responder	non-responder
VLDL	(0-40 mg/dl)	35 mg/dl (26/41)	32 mg/dl (22/42)
Lp(a)	(<30 mg/dl)	21.4 mg/dl (10/76)	12.8 mg/dl (10/49)
Triglycerides	(<150 mg/dl)	132.5 mg/dl (113/184)	128.5 mg/dl (90/168)
LDH	(<250 U/l)	209 U/l (167/257)	181 U/l (167/206)
Glucose	(74-106 mg/dl)	97.5 mg/dl (88/120)	99 mg/dl (89/132)
Vitamin D	(75-250 nmol/l)	61.89 nmol/l (+/-39)	41.8 mg/dl (+/-22)

hormonal metabolites	responder	non-responder
Estradiol (E2) (males)	0.14 nmol/l (0.11/0.16)	0.11 nmol/l (0.1/0.13)
SHGB	69.59 nmol/l (+/- 37.65)	56.43 nmol/l (+/- 21.64)
Cortisol	12.9 µg/dl (+/- 4.55)	11.9 µg/dl (+/- 3.74)
TSH	1.32 U/l (0.89/2.65)	1.39 U/l (1.19/2.18)
fT3	2.57 ng/l (+/- 0.44)	2.59 ng/l (+/- 0.28)
IGF-1	111 ng/ml (95.05/145.5)	132 ng/ml (107/162.6)
NT-proBNP	90.5 pg/ml (62.25/446.5)	251 pg/ml (106.5/412.5)

Depending on normality tests the mean with standard deviation (+/-) or median with (25%/75%) percentile is shown.
