# Supplementary material for: Immunometabolic Markers in a Small Patient Cohort Undergoing Immunotherapy
Source: Biomolecules. 2022 May 18;12(5):716. doi: 10.3390/biom12050716 (PMC9139165; doi:10.3390/biom12050716)
Supplement: Supplementary file 1 [file biomolecules-12-00716-s001.zip › Supplemental Data 5 ROC curves, cut off values and biomarker map statistics.pptx]

## Slide 1
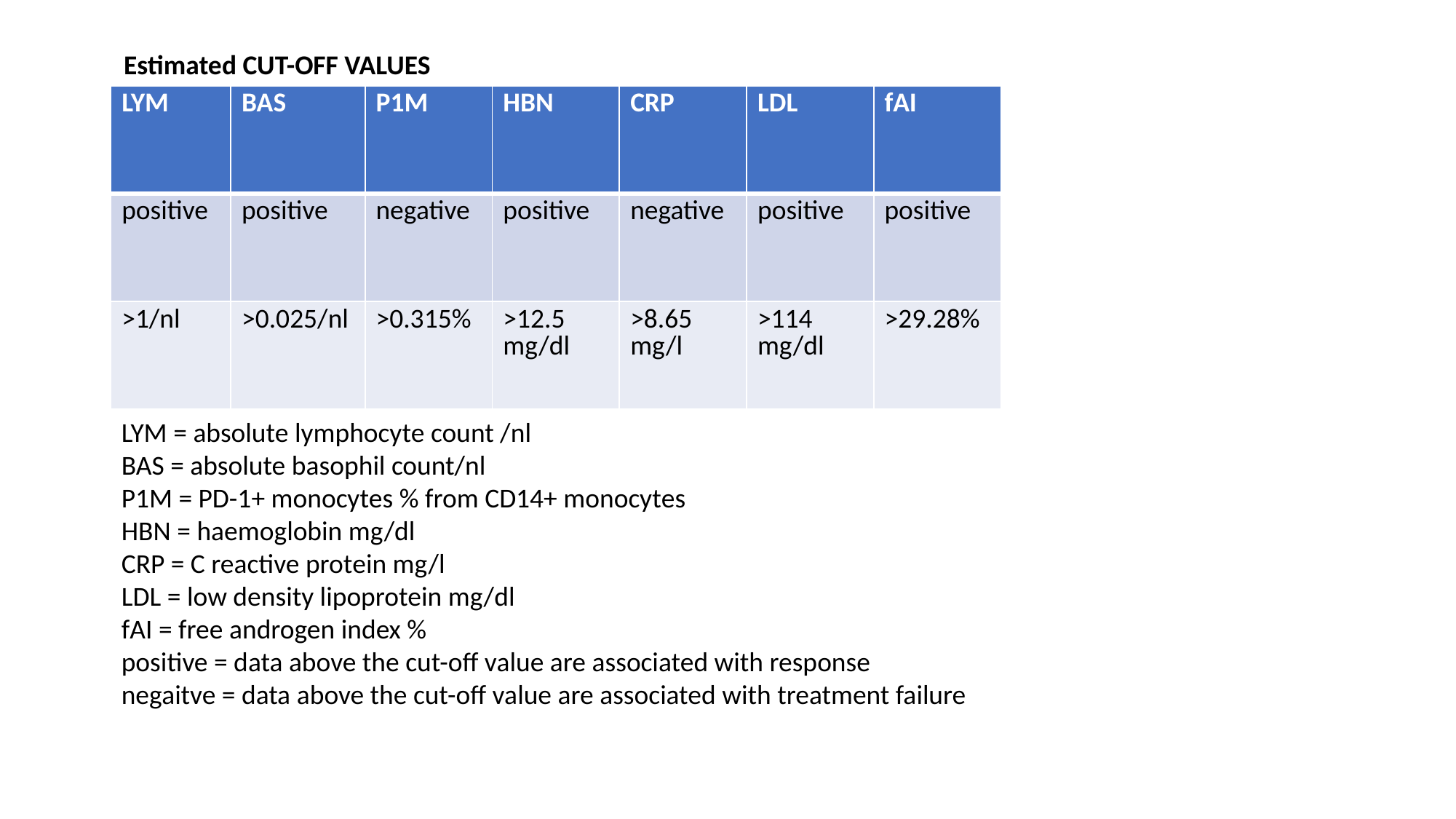

Estimated CUT-OFF VALUES
| LYM | BAS | P1M | HBN | CRP | LDL | fAI |
| --- | --- | --- | --- | --- | --- | --- |
| positive | positive | negative | positive | negative | positive | positive |
| >1/nl | >0.025/nl | >0.315% | >12.5 mg/dl | >8.65 mg/l | >114 mg/dl | >29.28% |
LYM = absolute lymphocyte count /nl
BAS = absolute basophil count/nl
P1M = PD-1+ monocytes % from CD14+ monocytes
HBN = haemoglobin mg/dl
CRP = C reactive protein mg/l
LDL = low density lipoprotein mg/dl
fAI = free androgen index %
positive = data above the cut-off value are associated with response
negaitve = data above the cut-off value are associated with treatment failure

## Slide 2
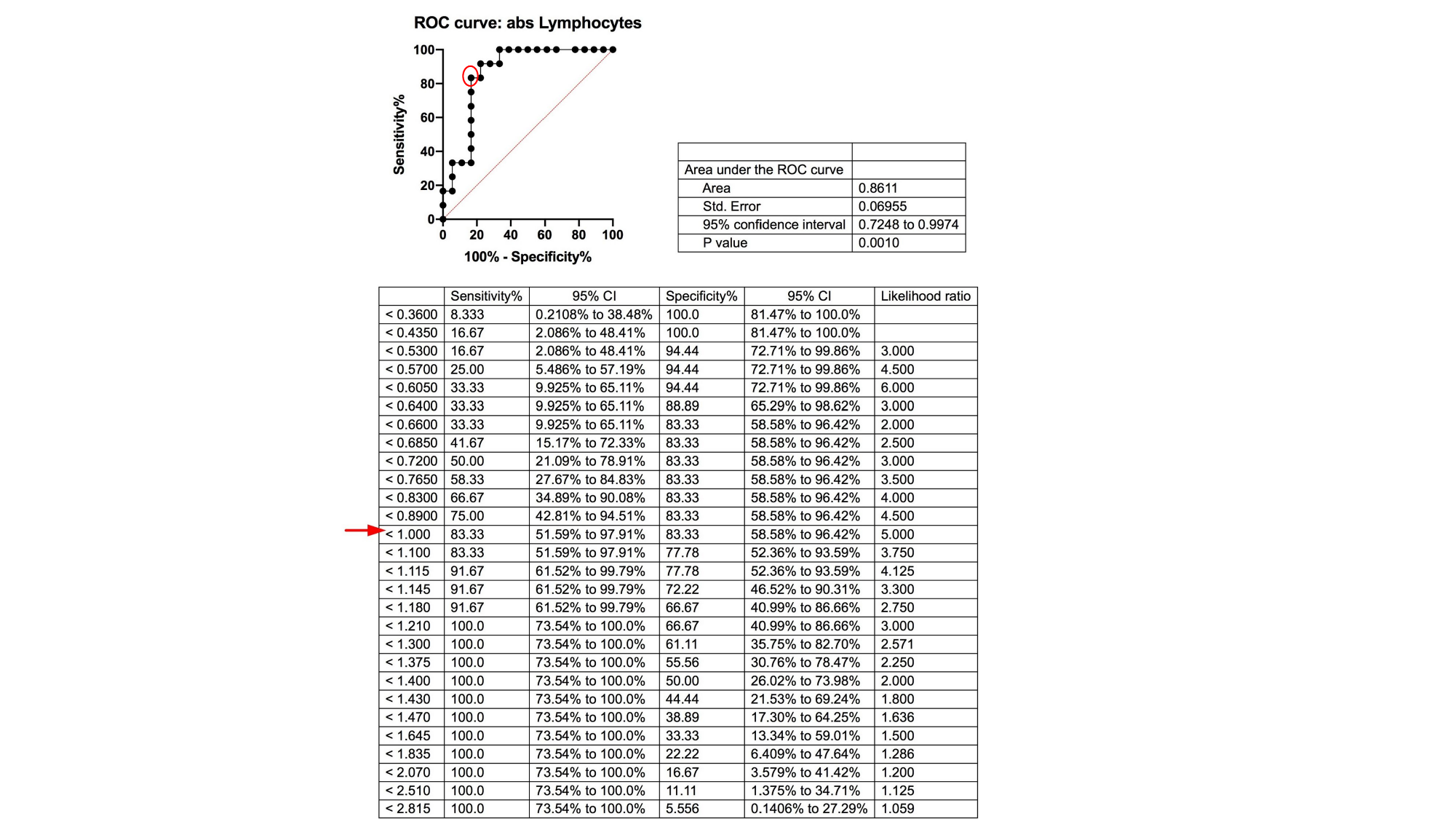

## Slide 3
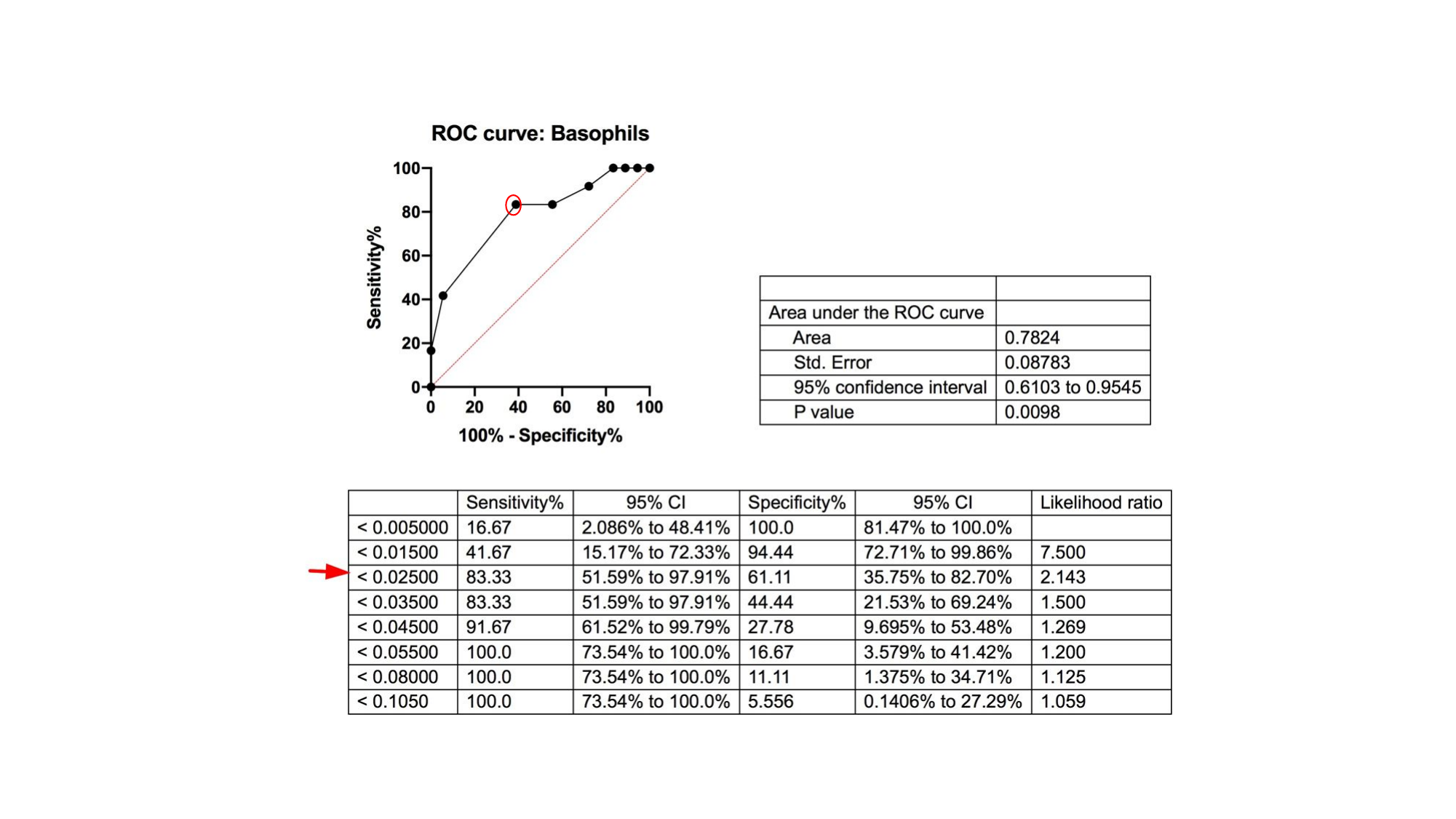

## Slide 4
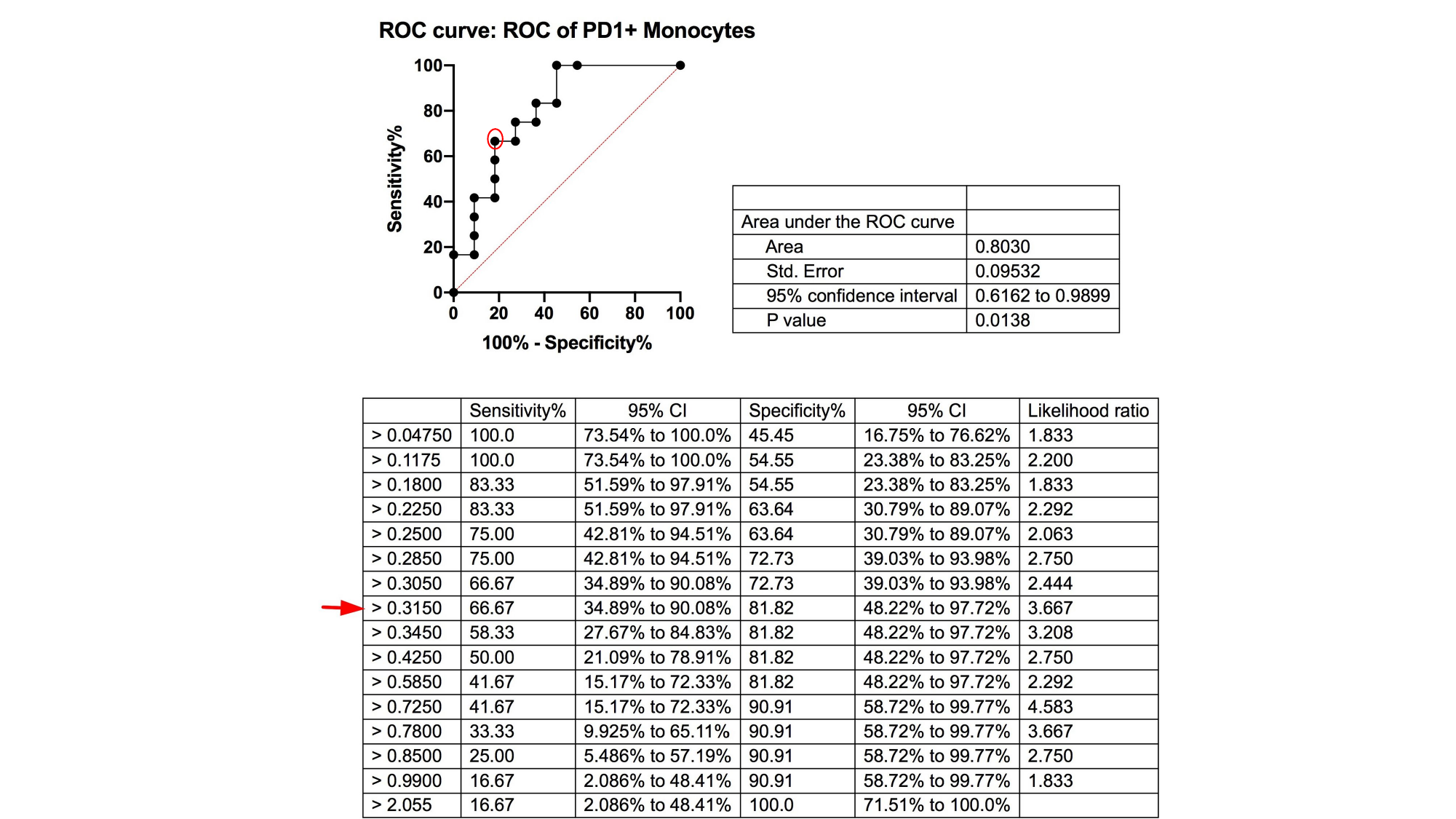

## Slide 5
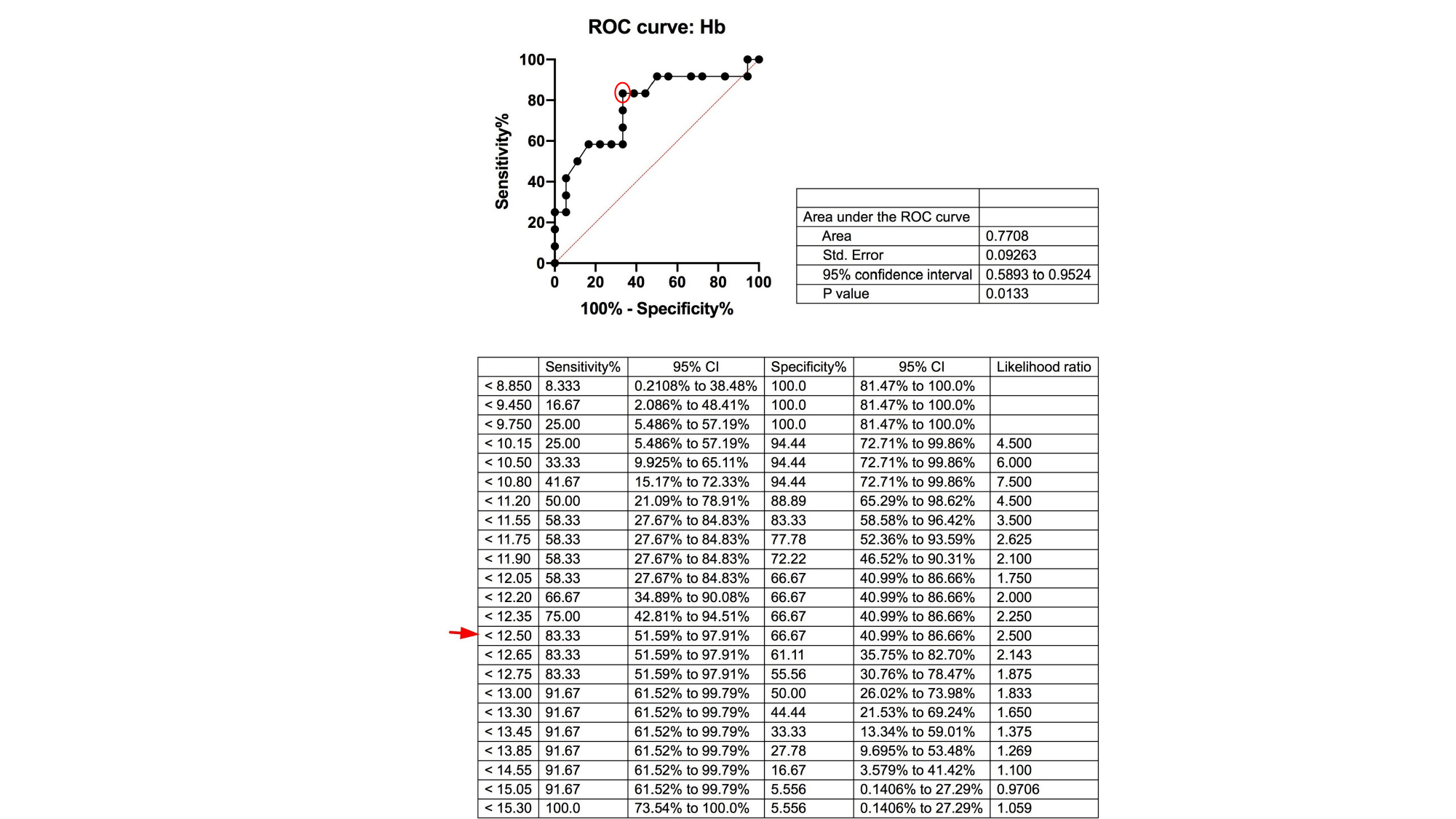

## Slide 6
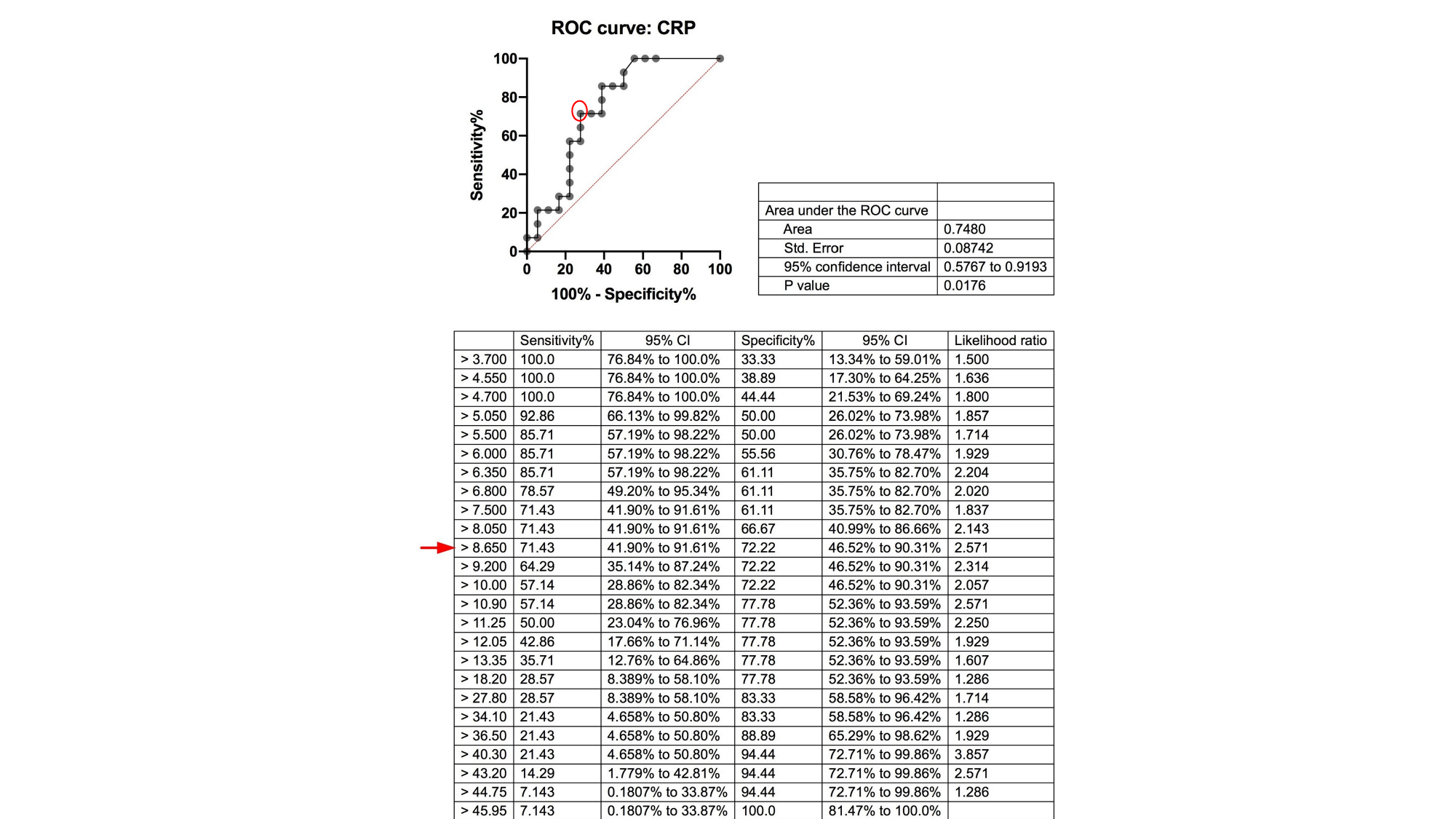

## Slide 7
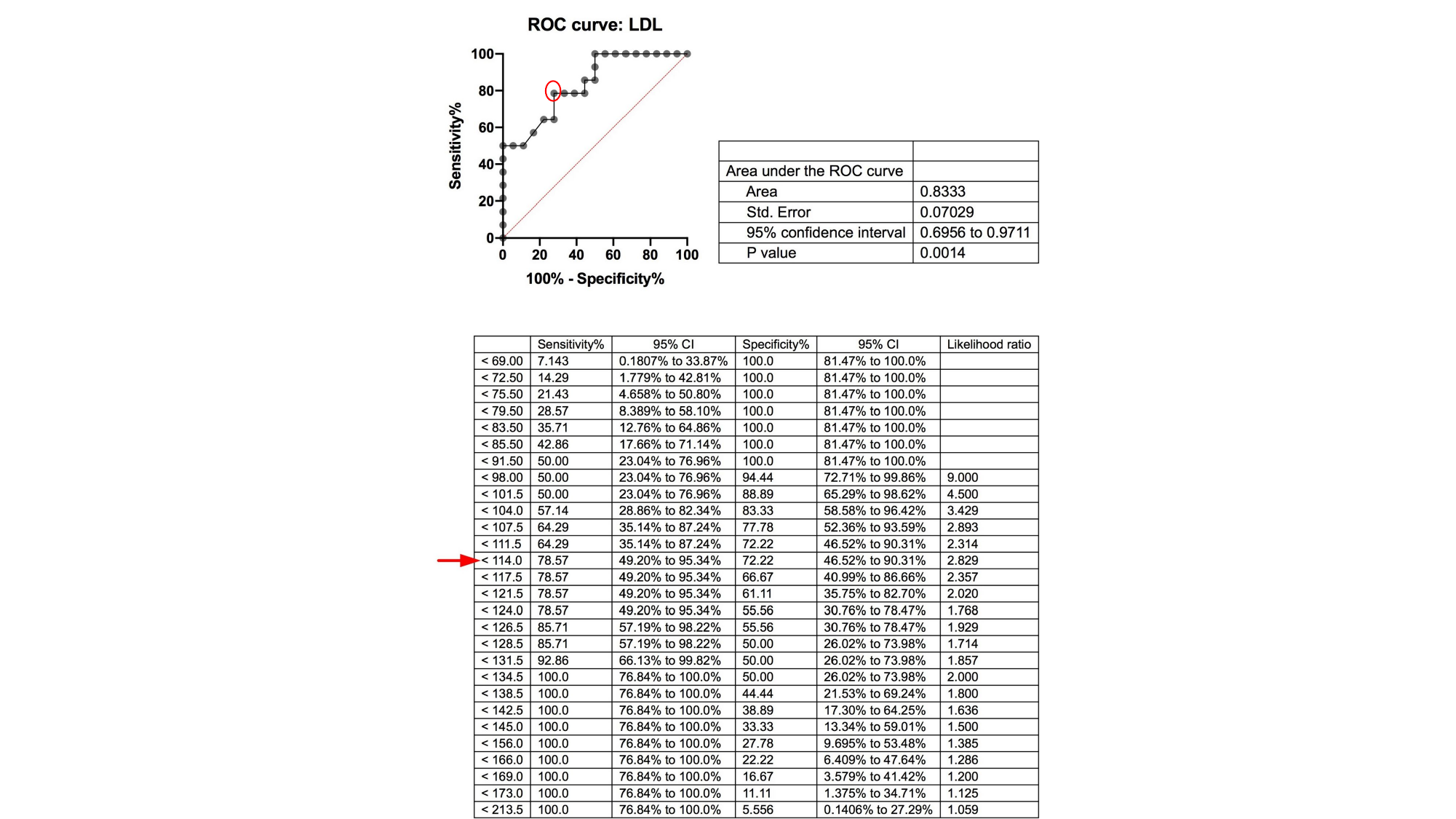

## Slide 8
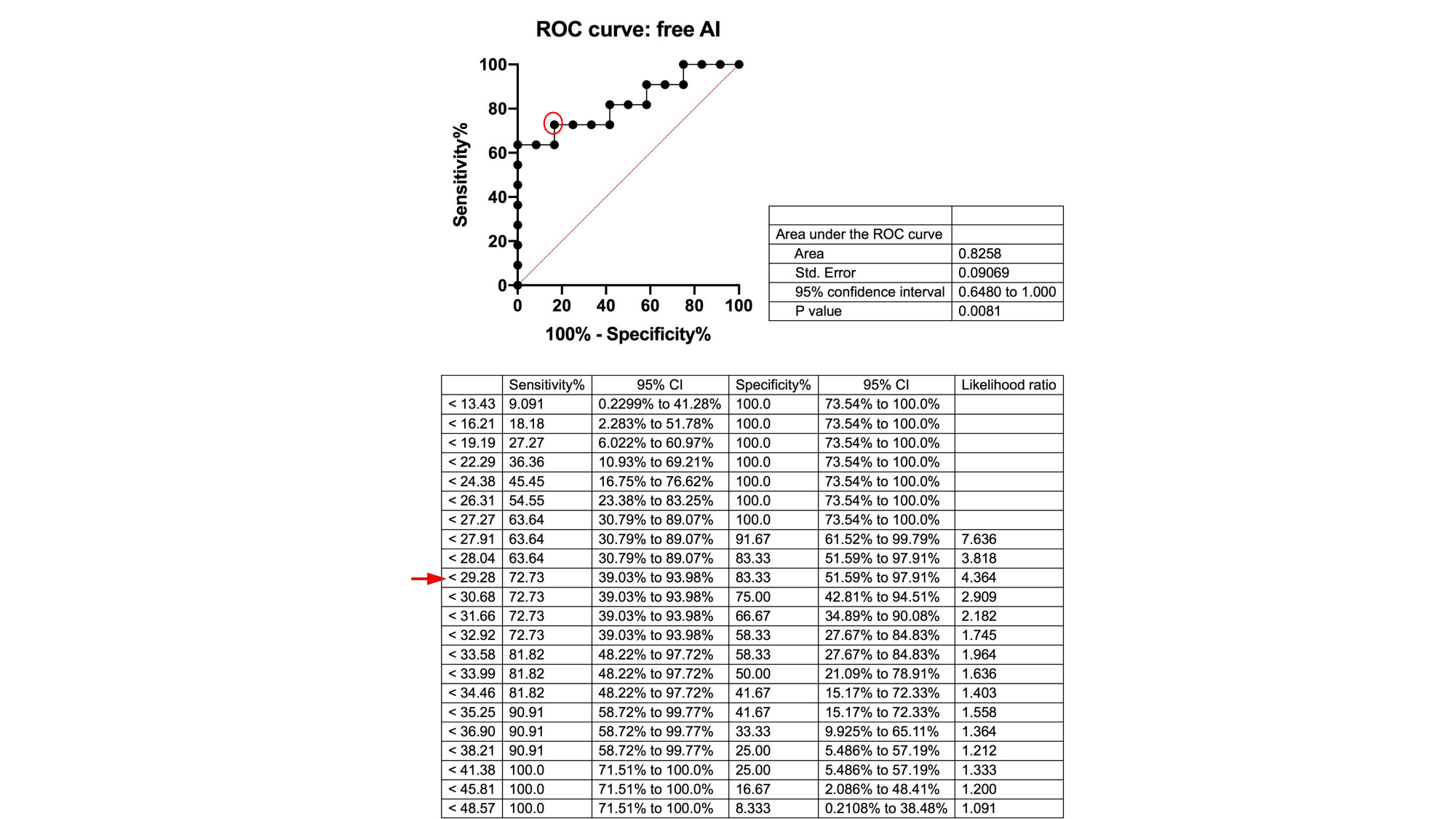

## Slide 9
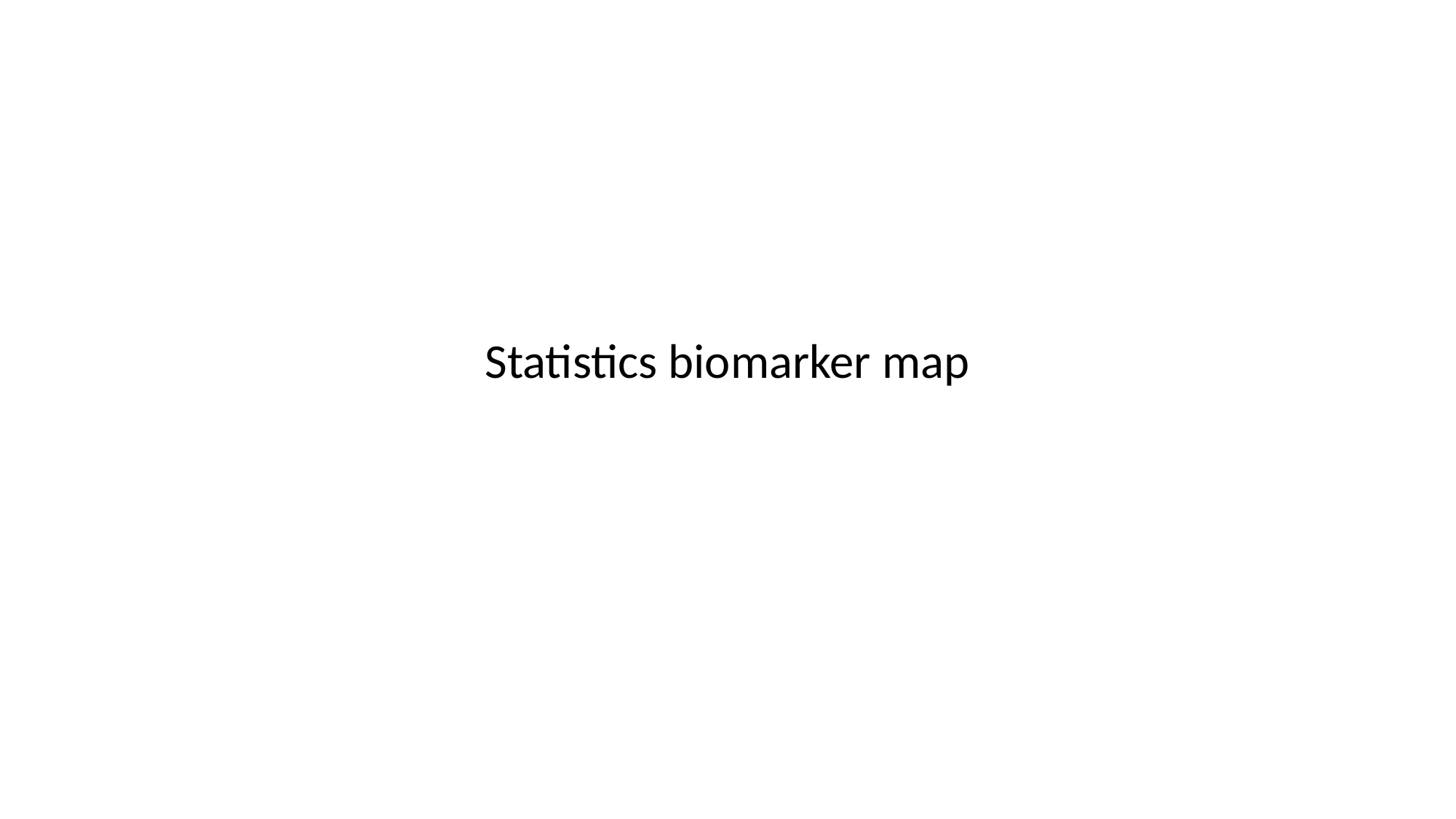

Statistics biomarker map

## Slide 10
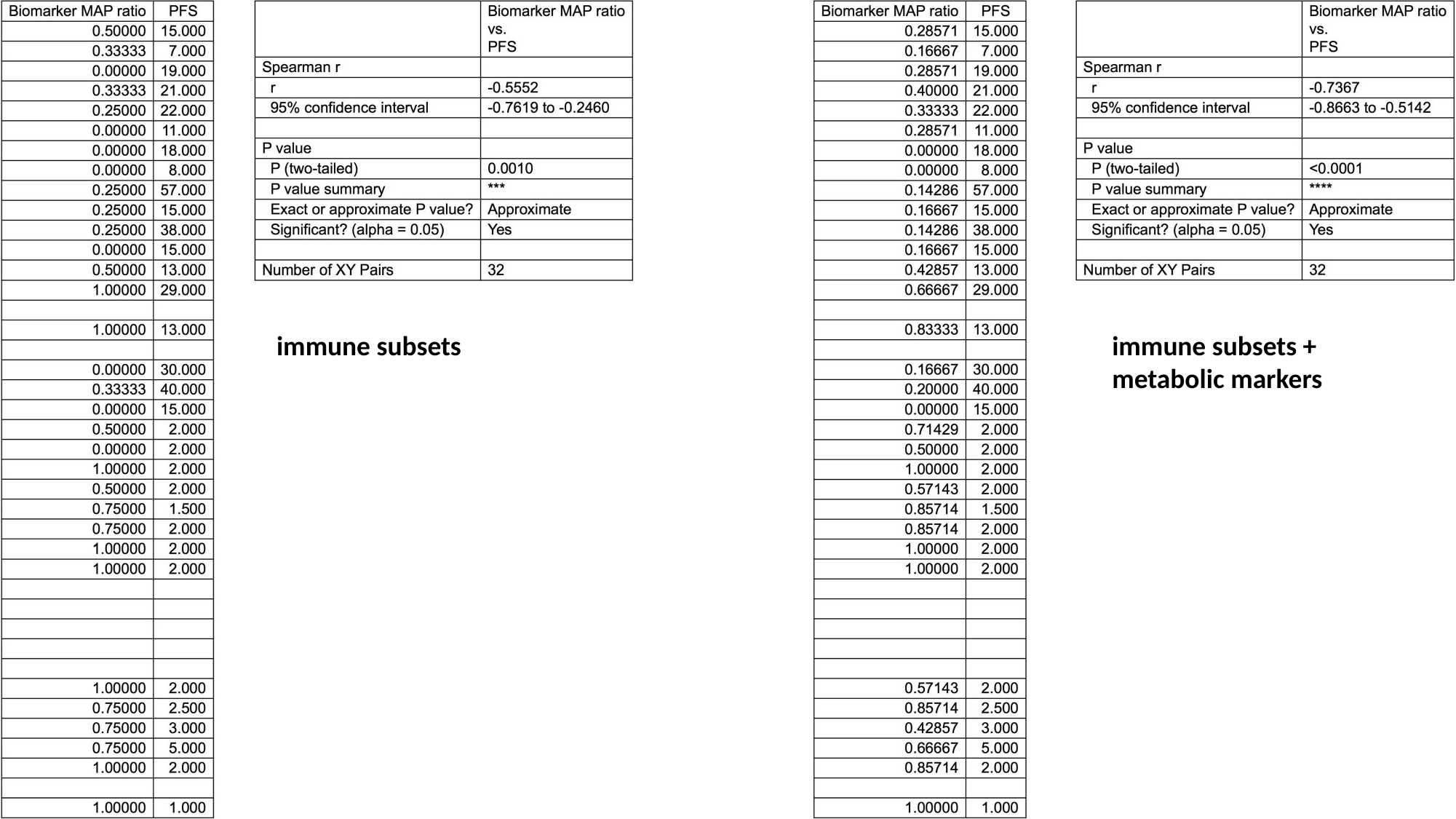

immune subsets
immune subsets +
metabolic markers
